# Associations of 6600 SomaScan proteins with demographic, lifestyle, environmental and health characteristics in Chinese adults

**DOI:** 10.1038/s41598-026-41444-z

**Published:** 2026-05-09

**Authors:** Ka Hung Chan, Jonathan Clarke, Maria G. Kakkoura, Andri Iona, Baihan Wang, Charlotte Clarke, Neil Wright, Pang Yao, Mohsen Mazidi, Pek Kei Im, Maryam Rahmati, Christiana Kartsonaki, Sam Morris, Hannah Fry, Iona Y. Millwood, Robin G. Walters, Yiping Chen, Huaidong Du, Ling Yang, Maxim Barnard, Dan Valle Schmidt, Yongmei Liu, Canqing Yu, Dianjianyi Sun, Jun Lv, Michael Hill, Liming Li, Robert Clarke, Derrick A. Bennett, Zhengming Chen

**Affiliations:** 1 Clinical Trial Service Unit, Nuffield Department of Population Health, Big Data Institute, University of Oxford, Old Road Campus, Oxford, OX3 7LF UK; 2 Department of Epidemiology & Biostatistics, School of Public Health, Peking University, Haidian, Beijing China; 3 Center for Public Health and Epidemic Preparedness and Response, Peking University, Haidian, Beijing China; 4 Key Laboratory of Epidemiology of Major Diseases (Peking University), Ministry of Education, Haidian, Beijing China; 5 NCDs Prevention and Control Department, Qingdao CDC, China

**Keywords:** Exposome, Sex, Age, Frailty, Lifestyle, Protein biomarkers, Biobank, Chinese, Biomarkers, Risk factors

## Introduction

Proteins play essential roles in all living cells, and an optimal balance of protein levels influence human health^1^. Previous studies of individual plasma proteins have identified relevance of many easily measured biomarkers for disease diagnosis and understanding of disease mechanisms, including troponin reflecting cardiac injury^2^, alanine transaminase (ALT) reflecting liver damage^3^, and C-reactive protein (CRP) reflecting systemic inflammation^4^. Recent advances in high-throughput proteomic assays now enable measurements of thousands of plasma proteins^5^, which permits a more comprehensive investigation on the molecular mechanisms underlying disease aetiology.

An estimated 70–90% of disease risk in adults could be attributed to non-genetic risk factors, collectively known as the “exposome”, which act through multiple complex biological pathways in disease pathogenesis, with plasma proteins being likely intermediate markers of exposure or adverse effects. Recent population-based proteomics studies have assessed the associations of proteins, assayed using Olink or SomaScan platforms, with several well-established risk factors (e.g., adiposity, ageing, and smoking) and their associated biological processes^6–10^. However, most previous studies were conducted in European populations^7–10^, and did not provide systematic evaluations of impact of a wider spectrum of non-genetic factors on the plasma proteome^11,12^. An “exposome-wide” study of the correlates of plasma protein levels in diverse populations can inform future research priorities and guide analytical approaches (e.g., adjustment for potential confounders when studying specific exposures).

We undertook an exploratory assessment of the associations of approximately 7000 SomaScan proteins with 37 major non-genetic risk factors across six main domains in approximately 2000 Chinese adults in the China Kadoorie Biobank (CKB). The main objectives of the present study were to (i) systematically explore the exposure profiles of approximately 7000 SomaScan proteins in 2000 Chinese adults; (ii) examine whether these association profiles vary by sex; and (iii) assess whether association profiles differ importantly between normalised and non-normalised SomaScan protein levels.

## Methods

### Study population and design

Details of the study design and participants characteristics of CKB have been reported elsewhere^11^. Briefly, CKB recruited ~ 512,000 adults aged 30–79 years from 10 geographically diverse areas in 2004–2008. At baseline, trained health workers administered a comprehensive laptop-based questionnaire and recorded physical measurements, including anthropometry and blood pressure, using regularly-calibrated instruments following standardised protocols. Desktop biochemical assays included random plasma glucose (RPG) and hepatitis B surface antigen (HBsAg). A 10-mL non-fasting (with time since the last meal recorded) blood sample was collected from each participant, and then processed and stored in liquid nitrogen. The present study involved 2,026 randomly selected subcohort participants who had no prior history of cardiovascular disease, originally sampled along with 1,951 cases of incident ischaemic heart disease (IHD) for a case-cohort study (eFigure 1)^6,13^. After excluding participants with missing data on relevant variables, the current study included 1,998 subcohort participants (1243 females, 755 males) for the main and sex-specific analyses.

The CKB was approved by the Ethical Review Committee of the Chinese Center for Disease Control and Prevention (Beijing, China) and the Oxford Tropical Research Ethics Committee, University of Oxford (Oxford, UK), and all participants provided written informed consent upon recruitment.

### Proteomic assays

Details of the proteomic assays in the CKB have been described elsewhere^6,13–16^. In brief, 60 µl plasma aliquots in 2D-barcoded microtubes of 3,977 participants were delivered to the Somalogic Laboratory in Colorado, USA for profiling using SomaScan Assay v4.1, which covers 7,596 slow off-rate modified aptamers (SOMAmers) as protein-binding reagents. After excluding SOMAmers binding non-protein targets or targets in non-human organisms, a total of 7,289 SOMAmers targeting 6,597 human proteins were included in the current study (some proteins were targeted by more than one SOMAmer). Samples were randomly aliquoted into 96-well plates (including 11 wells for external control samples, including 5 calibrator, 3 QC, and 3 buffer samples). The raw output of the SomaScan assay were standardised based on external control samples to account for variability in microarrays and variations within and between plates. With an optional procedure of adaptive normalisation by maximum likelihood (ANML) to an external reference, which controls for inter-sample variability, the results were provided in normalised and non-normalised values in relative fluorescence units (RFU)^17^. The output values were further natural log-transformed in the main analysis. The limit of detection (LOD) for SOMAmers were defined with external buffer samples. Quality control (QC) checks were conducted comparing the median of QC samples on each plate to the reference, with a cross-place QC indicator (pass/flag) assigned to each SOMAmer. The 7,289 SOMAmers targeting human proteins were mapped to proteins based on their UniProt IDs supplied by Somalogic. Details of individual proteins and their distributions by sex are shown in eTable 1.

### Selected baseline characteristics

From the baseline questionnaire and physical measurements, we identified 37 key characteristics from six broad categories: demographics (e.g., age, sex, study area), lifestyle (e.g., alcohol, smoking, diet, physical activity), environmental factors (e.g., air pollution, ambient temperature), health and wellbeing (e.g., medical history and mental health), clinical measurements (e.g., BMI, HBV, RPG) and female reproductive factors (e.g., age at menarche, age at menopause, parity) (eTable 2). These 37 exposures were carefully selected to represent key categories relevant to the study objectives, while minimizing redundancy and avoiding collinearity of associated exposures within the same category. We also derived composite indices for (i) healthy lifestyle (ranging from 0 to 5, with higher score indicating a healthier lifestyle), based on our previous publications, which takes into account smoking, alcohol intake, physical activity, dietary habits, and body shape^18–20^; and (ii) frailty, based on 28 variables on self-reported medical conditions, symptoms, signs, and physical measurements that capture different aspects of cumulative health status deficits as described previously^21,22^ (see derivation details in eTable 2).

### Statistical analysis

The analyses were restricted to 2,026 subcohort participants. We excluded individuals with missing proteomics data (n = 4), samples with hybridization control scale factor out of range (n = 7), and those with missing ambient temperature data (n = 20), leaving 1,998 sub-cohort participants (1243 females, 755 males) for the main overall and sex-specific analyses.

Selected baseline characteristics were examined by sex, directly standardised according to age and study areas of the original CKB population structure to facilitate comparison. The primary analyses used normalised SomaScan protein levels (ANML values). Multivariable linear regression was used to examine the associations of the 37 baseline characteristics with normalized plasma protein levels (in RFU), adjusting in the main models for age, age^2^, sex, study area, fasting time, fasting time^2^, outdoor temperature, outdoor temperature^2^ and plate ID^13,15^. The covariates were included because they showed associations with the baseline characteristics and/or protein levels in our preliminary exploratory investigations. In the case where a particular variable was the exposure of interest, we did not include it as a covariate in the model. We also assessed the independence of the observed associations with specific exposures by mutually adjusting for relevant exposure variables. For proteins targeted by multiple SOMAmers, the association with the more extreme p-values per exposure was selected, and directional consistency across all corresponding SOMAmers was assessed. As sensitivity analyses, we repeated analyses using on non-normalized protein levels to evaluate the impact of ANML, which might have attenuated meaningful inter-individual heterogeneity. We also examined the associations (with normalised protein levels) by three classes of protein abundance, defined according to the SomaScan-supplied dilution factor for each protein, as low- (2 × 10^–1^), moderate- (5 × 10^–3^), and high- (5 × 10^–5^) abundance.

Given the high correlation between many SOMAmers (or protein levels), we adopted the multiple testing correction approach described by Gadd et al.^23^ Specifically, we derived principal components to estimate the number of independent tests. For proteins, 1,186 principal components explaining 90% of the cumulative variance in 7,335 SOMAmers (eFigure 2 and eTable 3), and for exposures 21 components explaining 90% of the cumulative variance in the 32 exposures, with an additional 5 exposures for reproductive factors included seperatly^23^. We then applied a Bonferroni correction across all linear regression models, using a Bonferroni-adjusted p-value threshold of 0.05/(1186 × 26) = 1.62 × 10^−6^. All statistical analyses were performed using R version 4.1.2^24^ and packages ‘*tidyverse’* and ‘*ggplot2’*.

## Results

Of the 1998 participants included the mean baseline age was 50.8 (SD 10.5) years and 62.2% were females. Compared with females, males were more likely to smoke (63.3% vs 2.3%) and to drink alcohol (37.2% vs 2.6%) and, but similar prevalence of self-reported poor health and prior diseases, and slightly lower healthy lifestyle index (Table 1).

**Fig. 1:**
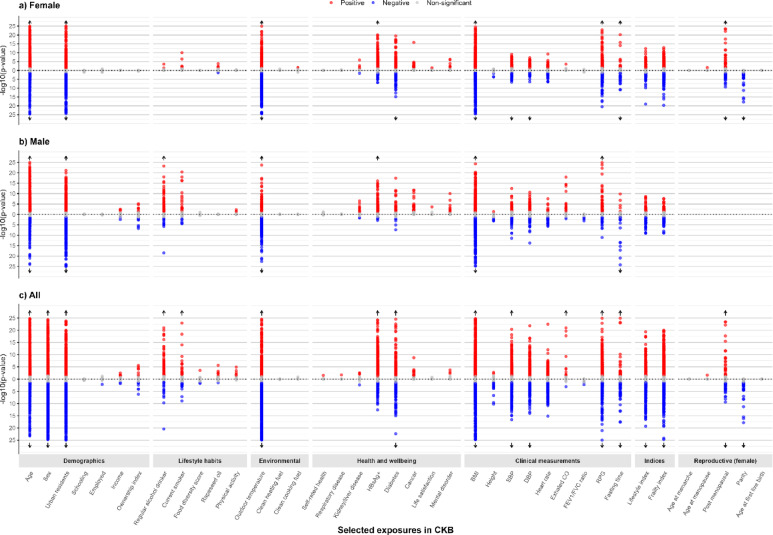
Exposure profile of 6597 SomaScan protein biomarkers in CKB, overall and by sex. Three Miami plots are presented: one for female-specific analysis, one for male-specific analysis, and one for overall analysis. The x-axis represents baseline characteristics grouped by category, while the y-axis shows the negative logarithm of the *p*-value (−log10 p-value) for the association between each exposure and protein biomarkers. Each dot represents the −log10 Bonferroni corrected *p*-value for these associations. For visualization purposes, −log10 *p*-values exceeding 25 are not displayed (indicated with arrow). Positive associations are shown in red, negative associations in blue, and non-significant associations in grey. Analyses performed based on ANML data. Analyses are adjusted for age, age^2^, sex, study area, fasting time, fasting time^2^, outdoor temperature, outdoor temperature^2^ and plate ID, where appropriate. Abbreviations: BMI: Body mass index; CKB: China Kadoorie Biobank; CO: carbon-monoxide; DBP: Diastolic blood pressure; FEV1/FVC: Forced Expiratory Volume in 1 s / Forced Vital Capacity; HBV: Hepatitis B virus; MET: metabolic equivalent task; RPG: random plasma glucose.

**Fig. 2:**
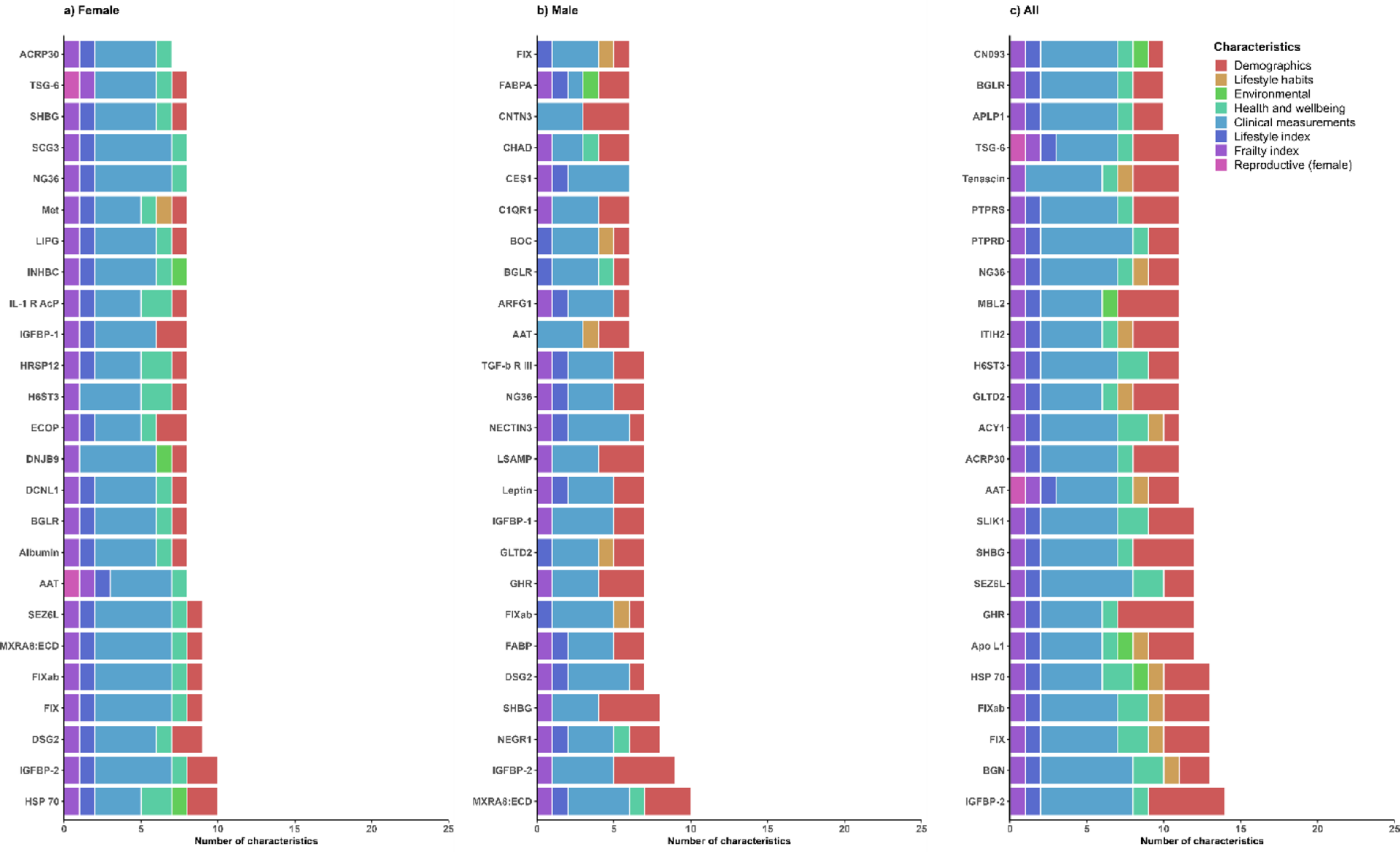
Exposure profiles of the top 25 SomaScan protein biomarkers with most associations, overall and by sex. The bar plots show the number of baseline associated with the 25 most frequently associated protein biomarkers after Bonferroni corrected *p*-value. The analyses are presented separately for females, males, and the overall. The x-axis represents the protein biomarkers, while the y-axis indicates the number of baseline characteristics associated with each protein. Bars are color-coded to represent different baseline characteristic groups. Analyses performed based on ANML data. Analyses are adjusted for age, age^2^, sex, study area, fasting time, fasting time^2^, outdoor temperature, outdoor temperature^2^ and plate ID, where appropriate.

Figure 1 shows the associations between exposures and proteins by their significance level, in all participants and by sex. Overall, 29 exposures were significantly associated with at least one protein in the main model after Bonferroni-adjustment, with 12 showing significant associations with > 50 proteins (Table 1; Fig. 1; eTable 4). In particular, sex (n = 996), age (n = 982), outdoor temperature (n = 802), and BMI (n = 1035) had the largest numbers of significant associations, followed by several clinical measurements (e.g. SBP and RPG), health and wellbeing indicators (e.g., HBsAg status and diabetes) and the lifestyle and frailty indices (Table 1; Fig. 1). In the mutually adjusted models the patterns of associations were similar, although there were fewer significant associations for sex (n = 593), age (n = 658), outdoor temperature (n = 751), BMI (n = 838), SBP (n = 38) and other exposures (eTable 5). In sex-specific analyses, there were more significant associations in females than in males, except for smoking (5 vs 24) and alcohol consumption (2vs 85), with three of the five female reproductive factors showing significant associations with 1–58 proteins (Table 1). Across the 6,597 unique human proteins from the 7,289 SOMAmers examined, 43% were associated with at least one exposure, with IGFBP-2 (n = 14), BGN (n = 13), FIX (n = 13), FIXab (n = 13), and HSP 70 (n = 13) associated with the greatest number of exposures, mostly with clinical measurements and demographic factors, overall and in sex-specific analyses (Fig. 2; eFigure 3).

**Fig. 3:**
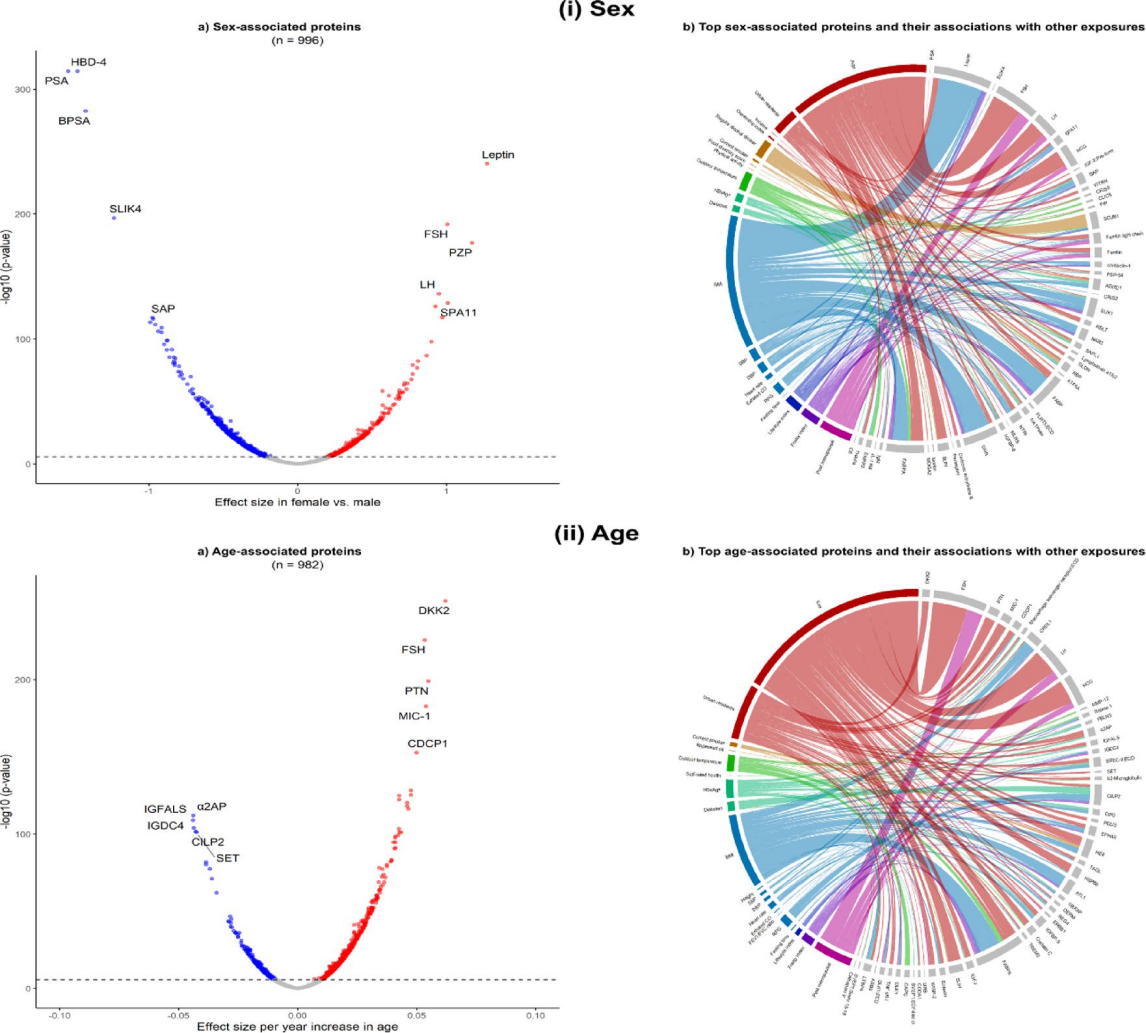
Sex- and age-associated SomaScan protein biomarkers and their associations with other exposures. Figures (**i**)(**a**) and (**ii**)(**a**) represent the associations of sex and age, respectively, with protein biomarkers. The x-axis represents the effect size of the association between sex or age and the protein biomarkers, while the y-axis indicates the –log10 *p*-value. Red dots denote positive Bonferroni corrected associations, blue dots denote negative Bonferroni corrected associations, and grey dots denote non-significant associations. Figures (**i**)(**b**) and (**ii**)(**b**) illustrate the top sex- and age-associated protein biomarkers, respectively, and their associations with other exposures. The width of the ribbons is inversely proportional to the *p*-value, indicating the strength of the association (smaller *p*-values correspond to wider ribbons). The colors of the ribbons represent different baseline characteristic groups. The top protein biomarkers that are not associated with other exposures are not presented in the figure. Analyses performed based on ANML data. Analyses are adjusted for age, age^2^, sex, study area, fasting time, fasting time^2^, outdoor temperature, outdoor temperature^2^ and plate ID, where appropriate. Abbreviations: BMI: Body mass index; CO: carbon-monoxide; DBP: Diastolic blood pressure; HBV: Hepatitis B virus; RPG: random plasma glucose.

Of the 996 proteins associated with sex, the most significant associations included higher levels of leptin, FSH, and PZP in females and higher levels of PSA, HBD-4, and BPSA in males (Fig. 3ia). Among the top 50 sex-related proteins, most were also associated with other exposures, particularly age (e.g., FSH, HCG) and BMI (e.g., leptin, FABP) (Fig. 3ib). Importantly, there were 180 proteins uniquely associated with sex, but not with any other exposures (eTable 6). Furthermore, apart from alcohol drinking and smoking where the exposure-protein associations were stronger in males (e.g. TBG, TINAL), most other associations appeared either comparable or slightly stronger in females (eFigure 4). The number of significant associations was also reduced in the mutually adjusted models among females (eTable 7) and males (eTable 8).

As for the 982 proteins associated with age, DKK2, FSH, PTN, MIC-1, and CDCP1 showed the most statistically significant positive associations, whereas α2AP, IGFALS, IGDC4, CILP2, and SET showed the most significant inverse associations (Fig. 3iia). In the mutually adjusted models, there was a reduction in the number of significant proteins (n = 658) (eTable 5). Of the top 50 (by statistical significance) age-related proteins, most were also associated with other exposures, particularly sex (e.g., FSH, PTN), BMI (e.g., CRDL1, FABPA), and urban residence (e.g., DKK2, MIC-1) (Fig. 3iib). In sex-specific analyses, age was associated with 735 and 593 proteins in females and males, respectively (Table 1), with most of the 357 overlapping proteins showing concordant associations (eFigure 5). Among the top age-associated proteins by sex, many were also associated with postmenopausal status, BMI, and urban residence in females, and with BMI and urban residence in males (eFigure 6). Consistently, the majority of the 58 proteins associated with postmenopausal status were also associated with age (e.g., FSH, LH, CRDL2, HCG), with some also being associated with BMI (e.g., FABPA, FABP) (eFigure 7).

HBsAg status and prevalent diabetes were associated with 468 and 257 proteins, respectively, but other health and wellbeing indicators, including self-rated health or other prior medical history showed few significant associations (Table 1). For HBsAg status, the top positively associated proteins were DHI1, NUD16, DJB12, C1QTNF3, and AKR1D1, while the top inversely associated proteins were CFHR5, SAP, RBP, IL-1 R AcP, and α2AP (eFigure 8). For prevalent diabetes, the top positively associated proteins included PLXB2 and SEMA6A and top inversely associated proteins were CILP2 and MXRA8:ECD (eFigure 8). In the mutually adjusted models, these associations were reduced for prevalent diabetes (n = 168), but increased for HBsAg positivity (n = 522) (eTable 5).

Several clinical measurements, including BMI, SBP, DBP, heart rate, and RPG (but not height, exhaled CO or lung function) were strongly associated with multiple proteins (Table 1), with the number of significant associations being reduced in the mutually adjusted models (eTable 5), particularly for SBP (n = 38) and RPG (n = 58). BMI had the highest number of associations (n = 1035) among all exposures examined, with leptin, GHR, FABP, FABPA, and GPDA being the top positively associated proteins and IGFBP-2, IGFBP-1, WFKN2, SHBG, and SEZ6L being the top inversely associated proteins (eFigure 9). In the sex-specific analyses, leptin, GHR, FABP, FABPA, IGFBP-2, WFKN2, and SHBG were also top hits with BMI, with the same direction of association in males and females (eFigure 10). The proteins that had the most significant positive associations with SBP were GHR, INHBC, FIXab, FIX, and leptin, while those with the most significant inverse associations were renin, IGFBP-2, SHBG, H6ST2, and SCG3 (eFigure 8). Among the top RPG-associated proteins, there were positive associations with PLXB2, SEMA6A, SEM4D, SEM6B, and NFASC and inverse associatons with CILP2, MXRA8:ECD, COL15A1, SCG3, and ALB (eFigure 8).

**Fig. 4:**
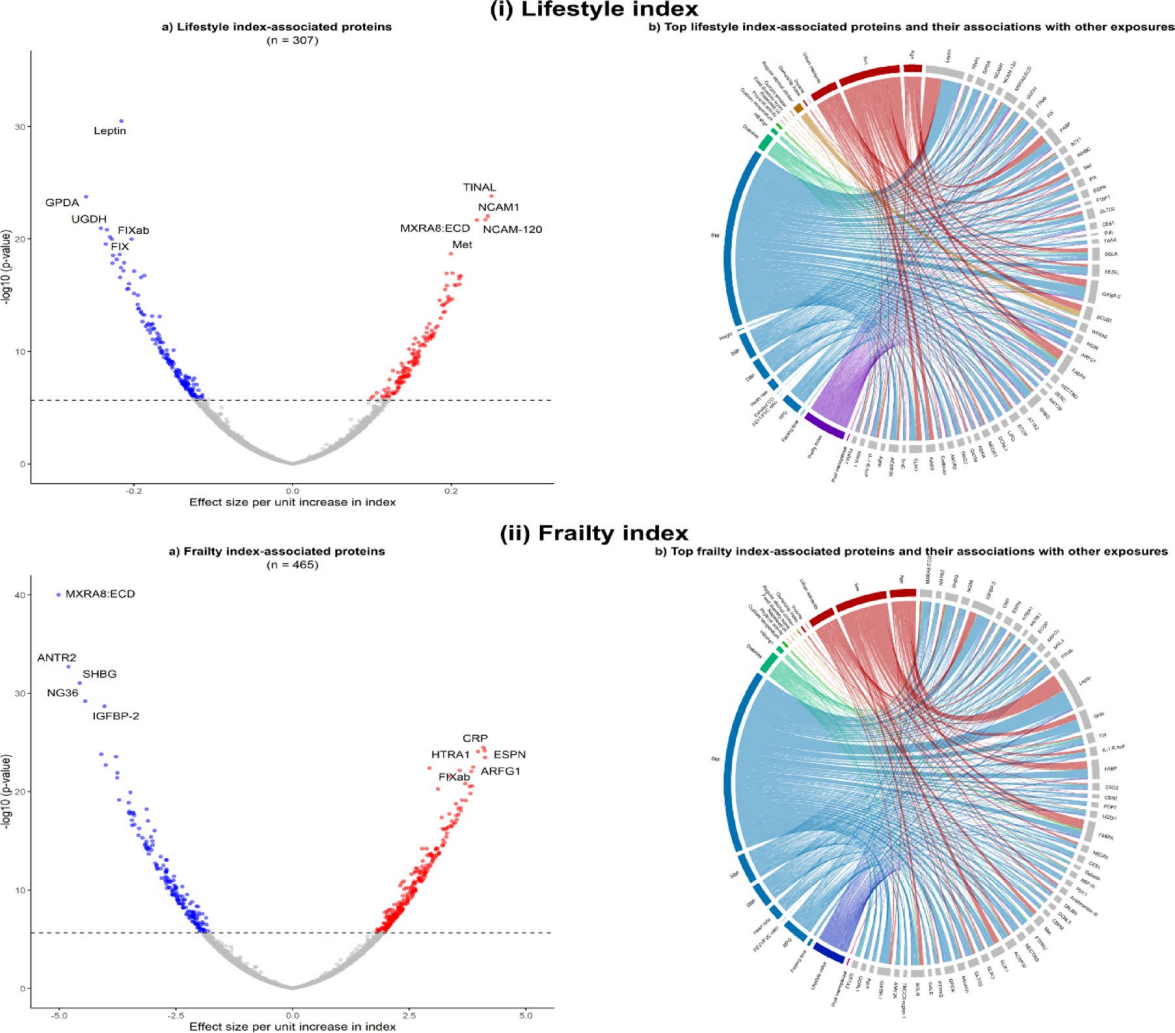
Lifestyle and frailty indices-associated SomaScan protein biomarkers and their associations with other exposures. Figures (**i**)(**a**) and (**ii**)(**a**) represent the associations of sex and age, respectively, with protein biomarkers. The x-axis represents the effect size of the association between sex or age and the protein biomarkers, while the y-axis indicates the –log10 *p*-value. Red dots denote positive Bonferroni corrected associations, blue dots denote negative Bonferroni corrected associations, and grey dots denote non-significant associations. Figures (**i**)(**b**) and (**ii**)(**b**) illustrate the top sex- and age-associated protein biomarkers, respectively, and their associations with other exposures. The width of the ribbons is inversely proportional to the *p*-value, indicating the strength of the association (smaller *p*-values correspond to wider ribbons). The colors of the ribbons represent different baseline characteristic groups. The top protein biomarkers that are not associated with other exposures are not presented in the figure. Analyses performed based on ANML data. Analyses are adjusted for age, age^2^, sex, study area, fasting time, fasting time^2^, outdoor temperature, outdoor temperature^2^ and plate ID, where appropriate. Abbreviations: BMI: Body mass index; CO: carbon-monoxide; DBP: Diastolic blood pressure; HBV: Hepatitis B virus; RPG: random plasma glucose.

Among the 307 proteins associated with healthy lifestyle index (Table 1), the most significant positive associations included TINAL, NCAM1, and NCAM-120, and the inverse associations included leptin, GPDA, and UGDH (Fig. 4ia). The index-protein associations, as illustrated in the most strongly associated proteins, appeared to overlap with those with BMI and sex, followed by clinical measurements (e.g., SBP, DBP, RPG), and urban residence (Fig. 4ib). The composite frailty index was associated with 465 proteins overall, and 226 and 82 in females and males, respectively (Table 1; Fig. 4iia). Among the leading frailty-associated proteins, there were positive associations with CRP, ESPN, and HTRA1 and inverse associations with MXRA8:ECD, ANTR2, and SHBG (Fig. 4iia). Importantly, the proteins most strongly associated with frailty index overlapped with most clinical measurements (particularly BMI), age, sex, and lifestyle index, in a largely coherent manner (i.e., opposing direction of association) as expected (Fig. 4iib). After mutual adjustments, the number of proteins significantly associated with the frailty index changed little (n = 440) (eTable 5).

Notably, we observed a general trend of higher proportion of significant associations with proteins of higher abundance (e.g., 41% proteins of high-abundance vs. 10% of low-abundance were associated with age), except for outdoor temperature (approximately 10% for both high- and low-abundance proteins) (eTable 9; eFigure 11). Analysis of non-normalised protein levels showed greater number (by 1.5 to 3 times) of significant associations with age, diabetes, BMI, SBP, DBP, heart rate, RPG, menopausal status, and lifestyle and frailty indices, but considerably fewer (15% lower) significant hits with outdoor temperature (eTable 10; eFigure 12). Generally, the proportional increment of significant associations in the non-normalised proteins were more prominent in females than males, except for BMI and frailty index (eTable 9). Importantly, the effect sizes of associations related to non-normalised protein levels were smaller than for normalised levels for age, BMI, SBP, RPG, prevalent diabetes, and frailty index, but the converse was true for lifestyle index, whereas the associations with sex were highly consistent (eFigure 13). Among proteins with Bonferroni significant associations and more than one nominally significant SOMAmer, > 80% showed directionally consistent associations with the selected SOMAmer for all 37 exposures considered (eTable 11).

Across the SomaScan and Olink platforms the analyses yielded comparable numbers of significant proteomic associations with the multiple exposures, particularly with sex, age, alcohol drinking, smoking, HBsAg status, diabetes, clinical traits, menopausal status, healthy lifestyle and frailty indices (eFigure 14).

## Discussion

This exposome-wide analyses of almost 6,600 SomaScan proteins in approximately 2,000 Chinese adults demonstrated significant associations of major non-genetic factors with plasma levels of specific proteins. Exposures such as age, sex, ambient temperature, and BMI demonstrated the largest number of significant and mostly independent associations with multiple proteins (600–840 proteins), with many of these associations varying by sex. Other exposures including demographic factors, lifestyle habits, clinical measurements, and lifestyle and frailty indices were also related to a very large number of proteins. Overall, there was a larger proportion of significant associations with high-abundance proteins, and this was even more pronounced in non-normalised SomaScan.

The two parallel analyses across the SomaScan and Olink^15^ platforms yielded largely consistent findings, especially on factors associated with large number of proteins. Notably, the patterns of associations with Olink proteins were more similar to those for the non-normalised SomaScan proteins, consistent with previous investigations. Moreover, both platforms showed directionally consistent associations, as demonstrated for instance by leptin (higher in females in both platforms) and IGFBP-2 (inversely associated with frailty index in both platforms). Furthermore, a number of proteins that are included in the SomaScan platform but not in the Olink platform were significantly associated with many exposures (e.g. age positively associated with DKK2, and frailty index inversely associated with ANTR2), demonstrating the complementary advantages of the two proteomic platforms.

Previous studies have investigated the SomaScan plasma proteomic profiles of various exposures^25–30^. However, they included primarily Western populations, used the earlier versions of the SomaScan platforms with fewer proteins measured, and typically focused on single or a small group of individual exposures^25–29^. The only recent broad-spectrum study that used an earlier SomaScan (v4.0) platform to explore the genetic and non-genetic predictors of 4,775 plasma proteins measured in approximately 8000 European adults, showed that non-modifiable factors, including genetic factors, age, and sex were the major determinants of plasma proteins (of 3,242 protein targets), which is somewhat in line with our findings (on age and sex). However, age in this European study was associated with a higher proportion of proteins than in our study, potentially due to larger sample size, the multiple testing approaches used (their FDR correction vs our more conservative Bonferroni correction across exposures and proteins), and potential underlying biological differences across populations. A few studies also used the earlier Olink platform assays to investigate the exposure profiles of approximately 90 proteins^31–33^. Our study is one of the first and largest proteomic-exposome profiles studies in East Asia that systematically evaluates the impact of a much wider spectrum of exposures on the plasma proteome.

Our analyses in the main and mutually adjusted models also identified important differences in proteomic-exposome profiles between males and females, including many proteins known to be involved in sex-specific biological processes. For example, FSH that stimulates the growth and development of follicles in females^34^, was higher in females than males, consistent with findings in the parallel CKB Olink proteomics study. Furthermore, levels of PZP, an immunosuppressive protein expressed by the placenta^35^, were higher in females than in males. while the levels of PSA/BPSA and HBD-4 , which are expressed in prostate and testes^36^, respectively, were higher in males. We also found leptin, which is known to play a key role in regulating energy balance and controlling body weight, to be significantly higher in females, reflecting the sex-specific variations in body composition measurements, as observed in the parallel CKB Olink proteomics study. Interestingly, adjustment for sex in our analyses did not attenuate the associations of the sex-related proteins with other exposures, possibly reflecting other sex-independent effects of the proteins. For example, FSH was also associated with age and leptin was associated with BMI, independent of sex. Therefore, these proteins might also be involved in biological processes (metabolic, inflammatory, or ageing processes) that could be influenced by exposures like age and BMI, beyond their associations with sex. Importantly, a previous study reported that although plasma levels of proteins differed substantially between sexes, only a small proportion of their protein quantitative loci showed sex differences (0.3% for protein targets included in SOMAscan assay v4)^37^. This suggests that these observed differences might be related mainly to the differential impact of non-genetic factors between sexes,^38^ and highlights the importance of performing sex-specific and sex-adjusted analyses in observational proteomic studies.

Importantly, some proteomic associations with individual lifestyle factors differed by sex. For example, TBG and sICAM-5, which were significantly associated in males, have been previously associated with alcohol intake and smoking^25,39^, respectively. These observed sex-differences likely reflect the markedly higher prevalence (and intensity) of alcohol drinking and smoking in men than in women in the Chinese population^40,41^, which is also consistent with the higher number of significant hits with alcohol drinking and smoking in males. Interestingly, there were limited proteomic associations with the other individual lifestyle factors, most notably diet and physical activity, which is consistent with the previous SomaScan proteomics-exposome study in Europeans. Lifestyle factors could influence the proteome dynamically and these dynamic protein changes are challenging to be captured fully by cross-sectional snapshots of protein levels^42^. However, it is worth noting that a recent study has reported associations between healthy dietary patterns and Olink proteins measured in the UK Biobank^43^. Although we were unable to examine dietary patterns in detail due to the limited sample size and the relatively crude dietary assessment methods used in the CKB which focused on key food groups. This pattern-based approach may be more useful than a single food diversity score for assessing the impact of diet on plasma proteins in future studies.

Age was most strongly and positively associated with DKK2, which is a glycoprotein known to regulate vertebrate development and to modulate the Wnt signalling pathway^44^. It is worth noting that DKK2 is a low-abundance protein that was not covered by the Olink Explore platform used in the parallel CKB study^15^. DKK2 has been found to be involved in the development of several ageing-related diseases, including cancer^45–47^. We also found older age to be strongly associated with higher levels of PTN, a secreted growth factor that regulates cellular proliferation, growth, differentiation and migration. Previous studies also reported higher levels of plasma PTN being significantly associated with chronological age^26,48^. Additionally, the protein that showed the strongest inverse association with age was α2AP, a serine protease inhibitor responsible for inactivating plasmin. Circulating plasmin is released in the plasma during fibrinolysis, which is an important regulator of blood coagulation process, and congenital deficiency of α2AP causes a rare bleeding disorder due to increased fibrinolysis, and an increased bleeding tendency with age in patients with heterozygous deficiency. We also found that many other age-related proteins were also independently associated with other risk factors. For example, CRDL1, a bone morphogenetic protein-4 antagonist, was associated with BMI, SBP and DBP. Moreover, in consistent with Olink proteins, we found numerous sex-specific top protein hits for age to be also associated with postmenopausal status, including FSH, which is known to be higher in menopausal women.

We found a large number of proteins associated with several clinical as well as health and wellbeing-related traits. BMI was associated with the largest number of significant associations with proteins (n = 838) in the mutually adjusted model, which aligns with the relatively high protein variance attributable to measure of body composition reported previously in Europeans. A number of these associations were previously evident in both observational and genetic analyses, such as the positive association with leptin and FABP, which is involved in the uptake, metabolism and transport of long-chain fatty acids^6,28^. Future studies should also consider assessing the associations of SomaScan proteins with central adiposity (e.g. waist circumference, waist-to-hip ratio), as it is known to be more strongly associated with risk of cardio-metabolic disease^49,50^ and with Olink proteins^16^, compared with BMI. Prevalent diabetes and RPG were both positively associated with PLXB2 (receptor for semaphorins involved in cell–cell signalling) and inversely associated with CILP2 (involved in cartilage scaffolding), which are supported by prior observational^51,52^ and genetic analyses^29,53^. In addition to many new findings that need to be explored further in subsequent studies, we also identified some previously reported associations, such as SBP with renin (enzyme involved in the renin–angiotensin–aldosterone system)^54^ in both SomaScan and Olink studies, and HBV infection with SAP (part of the innate immune humoral arm)^55^.

The present study identified a large number of proteomic associations with both the frailty^21^ and healthy lifestyle indices^18,19^. The two indices capture essentially opposing dimensions of health status, and many proteins showed opposing associations across the two indices, including HTRA1, MXRA8:ECD, ANTR2, leptin, GPDA, UGDH, IGFBP-2, and SHBG. HTRA1 is implicated in inhibiting active TGF-β, which is an anti-inflammatory cytokine^56^, while MXRA8:ECD and ANTR2 are both involved in angiogenesis. GPDA was previously shown to be positively associated with current smoking and alcohol drinking in the Framingham Heart Study^25^, which is line with our finding of an inverse association of lifestyle index with levels of this protein. Both GPDA and UGDH have also been implicated in multiple cancer types, and considered as possible therapeutic targets for treatment of cancer^57,58^. In addition, consistent with our findings related to the two indices, circulating IGFBP-2 and SHBG were reported to be inversely associated with obesity^59,60^, while a previous study showed lower circulating levels of IGFBP-3 (same protein family with IGFBP-2) to be associated with current smoking. Both IGFBP-2 and SHBG are involved in cancer and metabolic/reproductive system disorders, respectively, and they have also been identified in the Olink CKB study. Many indices-related proteins were also associated with individual exposures, particularly BMI, other clinical measurements, sex, and age, which is an expected finding as a number of these exposures are constituents of the indices. However, in the mutually adjusted model the number of significant proteins was reduced by approximately 100 proteins for lifestyle index, but not frailty index, suggesting that most of these associations were driven mainly by individual components of exposure.

Among the many exposures examined previously, the impact of outdoor temperature on the plasma proteome has been understudied^31,61^. Given the influence of non-optimal outdoor temperature on mortality, proteomics may help us better understand its impact on human health and clarify underlying mechanisms^62^. A recent analysis conducted in CKB identified nearly 1,000 Olink proteins associated with non-optimal ambient temperatures (cold and heat) and implicated pathways related to inflammation, immunity and platelet activation, highlighting the value of proteomics for understanding temperature-related health effects^63^. Here, we found a large number novel significant associations with SomaScan proteins that warrant further investigation. For example, SPINK6, which showed a strong positive association with ambient temperature, is a potent inhibitor of serine proteases that are essential for influenza A viruses infection in the airways^64^. Similarly, LRP1 and MMP7, which are inversely associated with temperature, play important roles in lipid metabolism^65,66^. However, we found fewer significant proteomic associations of proteins with several other exposures, including household air pollution, prevalent diseases (except diabetes), mental health, height, lung function and most reproductive factors. Future proteomic studies with a larger sample size and increased proteome coverage should help to further clarify the relevance of the observed associations and to identify new associations^42^.

We found that use of non-normalised proteomics panels yielded an even larger number of significant associations with several major exposures (e.g., age, diabetes, and frailty and healthy lifestyle indices). This is consistent with the previous report in CKB and with some previous studies using different approaches to proteomic profiling^67–69^. The normalization procedure adopted by SomaLogic standardises the overall signal of each sample to an external reference to improve data quality, which might be beneficial in removing false positives introduced by noise in the non-normalised data^70^. However, this process may also remove true extreme signals that are present in the general population, attenuating the number of observed significant associations^67,71^. Interestingly, while most associations with the two types of data were directionally consistent, the effect sizes of the overlapping associations were smaller for non-normalised than for normalised protein levels.

The present study is the first and largest study in East Asia that measured the levels of 6,600 plasma proteins using the SomaScan platform. Our “exposome-wide” investigation, in parallel with that on 2923 Olink proteins, can inform future analytical approaches (e.g. confounding adjustment) when examining specific exposures. Moreover, 65% of proteins covered by the SomaScan v4.1 platform were not included in the Olink Explore platform also used in CKB, with 87% of these non-overlapping proteins being low-abundance, which may be equally important for revealing exposure-protein associations. The analysis of both high- and low-abundance proteins provide hypotheses for future studies to investigate their underlying biological processes, which may lead to insights into disease pathogenesis and progression. However, our study also had limitations. First, there were more females than males, which might bias towards finding a larger number of significant associations in females. Moreover, there was limited statistical power for performing further subgroup analyses, besides the sex-specific analyses. Second, the present study was restricted to proteins measured only in plasma, which only cover a modest proportion of the human proteome. Third, replication of our findings in external and independent cohorts was not possible, as currently there are limited available SomaScan proteomic data in other East Asian populations. Nevertheless, our findings are broadly consistent with the parallel analyses on Olink proteomics which included 2,168 proteins also targeted by SomaScan. Fourth, the direction of the associations studied herein could not be confirmed due to the cross-sectional nature of the study. Fifth, although the analyses were adjusted for a range of key covariates, such as age, sex, region, and fasting time, residual confounding cannot be fully excluded and causality could not be reliably established. Potential non-linear exposure–protein relationships may also not have been fully captured, which should be explored further in future studies focused on specific exposures. Sixth, given the descriptive, hypothesis-free nature of the study, we did not undertake genetic analyses, which could help clarify potential causal relevance between exposures and proteins^72^. Further functional analyses of proteins associated with a specific exposure would also be beneficial in clarifying the underlying biological pathways involved, although this is beyond the scope of the current study.

Overall, this large proteomic study in the Chinese population showed that several exposures, particularly, age, sex, ambient temperature, BMI and composite indices of healthy lifestyle and frailty were associated with a large number of proteins that are involved in multiple pathways. The associations between major exposures and proteins also varied by sex, potentially due to sex-specific biology and sex-differential lifestyles. Analyses using non-normalised SomaScan data yielded a greater number of significant associations, consistent with the possibility that normalisation may attenuate some genuine inter-individual variation. More studies using the SomaScan platform in East Asian cohorts, are warranted to replicate the current findings. Although large proteomic datasets in East Asian populations are still limited, a parallel CKB study using the Olink platform in the same participants provides supportive evidence for reproducibility of these findings^15^. Studies using other alternative platforms in diverse populations will also be beneficial to assess the generalisability of the reported exposure-protein associations. Genetic analyses, such as Mendelian Randomisation, may provide additional information on the causal relevance of these associations and their underlying biological mechanisms.

## Supplementary Information

Below is the link to the electronic supplementary material.

Supplementary Material 1

Supplementary Material 2

## Data Availability

Data from baseline resurveys, and disease follow-up are available for access by bona fide researchers. Details of the CKB Data Sharing Policy, data release schedules and data request application procedures are available at [www.ckbiobank.org] . All queries about data access can be made to ckbaccess@ndph.ox.ac.uk.

## Abbreviations

AKR1D1: 3-Oxo-5-beta-steroid 4-dehydrogenase
ALT: Alanine aminotransferase
ANML: Adaptive normalisation by maximum likelihood
ANTR2: Anthrax toxin receptor 2
APOF: Apolipoprotein F
BGN: Biglycan
BMI: Body mass index
BPSA: Benign prostatic-specific antigen
C1QTNF1: C1q and tumor necrosis factor related protein 1
C1QTNF3: C1q and tumor necrosis factor related protein 3
CFHR5: Complement factor H-related 5
CILP2: Cartilage intermediate layer protein 2
CKB: China Kadoorie biobank
CO: Carbon monoxide
CRDL1: Chordin-like protein 1
CRDL2: Chordin-like protein 2
CRP: C-reactive protein
DBP: Vitamin D-binding protein
DKK2: Dickkopf-related protein 2
DJB12: DnaJ homolog subfamily B member 12
ESPN: Espin
FABP: Fatty acid binding protein
FABPA: Fatty acid binding protein, adipocyte
FIX: Coagulation factor IX
FIXab: Coagulation factor IXab
FSH: Follicle stimulating hormone
GHR: Growth hormone receptor
GPDA: Glycerol-3-phosphate dehydrogenase [NAD( +)], cytoplasmic
HBD-4: Hemoglobin subunit delta-4
HBsAg: Hepatitis B surface antigen
HBV: Hepatitis B virus
HCG: Human chorionic gonadotropin
HTRA1: Htra serine peptidase 1
H6ST2: Heparan-sulfate 6-O-sulfotransferase 2
DHI1: Corticosteroid 11-beta-dehydrogenase isozyme 1
IGDC4: Immunoglobulin superfamily, dcc subgroup member 4
IGFALS: Insulin-like growth factor binding protein, acid labile subunit
IGFBP-2: Insulin-like growth factor binding protein 2
IGFBP-3: Insulin-like growth factor binding protein 3
IHD: Ischemic heart disease
IL-1 R AcP: Interleukin-1 receptor accessory protein
INHBC: Inhibin beta C chain
LH: Luteinizing hormone
LOD: Limit of detection
LPL: Lipoprotein lipase
LRP1: Ldl receptor related protein 1
MIC-1: Macrophage inhibitory cytokine 1
MMP7: Matrix metallopeptidase 7
MXRA8: Matrix remodeling associated 8
MXRA8:ECD: Matrix remodeling associated 8, extracellular domain
NCAM1: Neural cell adhesion molecule 1
NCAM-120: Neural cell adhesion molecule 120 kda isoform
NFASC: Neurofascin
NUD16: U8 snorna-decapping enzyme
PLXB2: Plexin B2
PSA: Prostate-specific antigen
PTN: Pleiotrophin
PZP: Pregnancy zone protein
QC: Quality control
RBP: Retinol-binding protein 4
RFU: Relative fluorescence units
RIDA: Rectal intestinal domain antigen
RPG: Random plasma glucose
SAP: Serum amyloid P-component
SBP: Systolic blood pressure
SCG3: Secretogranin-3
SEMA6A: Semaphorin-6A
SEM4D: Semaphorin-4D
SEM6B: Semaphorin-6B
SD: Standard deviation
SHBG: Sex hormone-binding globulin
sICAM-5: Soluble intercellular adhesion molecule 5
SOMAmer: Slow off-rate modified aptamers
SPINK6: Serine peptidase inhibitor, kazal type 6
TBG: Thyroxine-binding globulin
TINAL: Tubulointerstitial nephritis antigen-like 1
TTR: Transthyretin
UGDH: Udp-glucose 6-dehydrogenase
WFKN2: WAP, Kazal, immunoglobulin, Kunitz and NTR domain-containing protein 2
α2AP: A2-antiplasmin
