# Supplementary material for: Associations of 6600 SomaScan proteins with demographic, lifestyle, environmental and health characteristics in Chinese adults
Source: Sci Rep. 2026 May 9;16:21236. doi: 10.1038/s41598-026-41444-z (PMC13346620; doi:10.1038/s41598-026-41444-z)
Supplement: Supplementary file 1 — Supplementary Material 1 [file 41598_2026_41444_MOESM1_ESM.docx]

**Supplementary Material**

**Associations of 6600 SomaScan proteins with demographic, lifestyle, environmental and health characteristics in Chinese adults**

Ka Hung Chan^1*^, Jonathan Clarke^1*^, Maria G Kakkoura^1*^, Andri Iona^1^, Baihan Wang^1^, Charlotte Clarke^1^, Neil Wright^1^, Pang Yao^1^, Mohsen Mazidi^1^, Pek Kei Im^1^, Maryam Rahmati^1^, Christiana Kartsonaki^1^, Sam Morris^1^, Hannah Fry^1^, Iona Y Millwood^1^, Robin G Walters^1^, Yiping Chen^1^, Huaidong Du^1^, Ling Yang^1^, Maxim Bernard^1^, Dan Valle Schmidt^1^, Canqing Yu^2,3,4^, Dianjianyi Sun^2,3,4^, Jun Lv^2,3,4^, Michael Hill^1^, Liming Li^2,3,4^, Robert Clarke^1^, Derrick A Bennett^1†^, Zhengming Chen^1†^, on behalf of China Kadoorie Biobank Collaborative Group^#^

1. Clinical Trial Service Unit, Nuffield Department of Population Health, University of Oxford, Oxford, UK
2. Department of Epidemiology & Biostatistics, School of Public Health, Peking University, Beijing, China
3. Peking University Center for Public Health and Epidemic Preparedness and Response, Beijing, China
4. Key Laboratory of Epidemiology of Major Diseases (Peking University), Ministry of Education, Beijing, China

** Co-first author;* ^†^Co-corresponding author; *# Members of the CKB Collaborative Group are shown in the Appendix.*

**Address for correspondence:**

Professor Derrick Bennett or Professor Zhengming Chen

CTSU, Big Data Institute, CTSU, Big Data Institute,

Old Road Campus, Old Road Campus

University of Oxford University of Oxford

Oxford, OX3 7LF, UK Oxford, OX3 7LF, UK

Tel: 44-1865-743949 Tel: 44-1865-743839

d[errick.bennett@ndph.ox.ac.uk](mailto:errick.bennett@ndph.ox.ac.uk) zhengming.chen@ctsu.ox.ac.uk

9 December 2025

**Contents**

[Members of the China Kadoorie Biobank Collaborative Group 4](#_Toc215826024)

[eTable 1. List of ~7300 SOMAmers targeting human protein biomarkers quantified by the SomaScan Assay v4.1 5](#_Toc215826025)

[eTable 2. Baseline characteristics and description of comparison used in analyses 6](#_Toc215826026)

[eTable 3. Principal component analysis for SomaScan protein biomarkers and baseline characteristics 8](#_Toc215826027)

[eTable 4. Effect estimates from the association of baseline characteristics with proteins 9](#_Toc215826028)

[eTable 5. Number of proteins significantly associated with baseline characteristic after sequential adjustments, in overall analyses 10](#_Toc215826029)

[eTable 6. Number of SomaScan proteins significantly associated with only one baseline characteristic, overall and by sex, in normalized and non-normalised datasets 11](#_Toc215826030)

[eTable 7. Number of proteins significantly associated with baseline characteristic after sequential adjustments, in females 12](#_Toc215826031)

[eTable 8. Number of proteins significantly associated with baseline characteristic after sequential adjustments, in males 13](#_Toc215826032)

[eTable 9. Number of SomaScan proteins significantly associated with baseline characteristics by abundance level 14](#_Toc215826033)

[eTable 9. Number of SomaScan proteins significantly associated with baseline characteristics, in normalised and non-normalised datasets 15](#_Toc215826034)

[Table 11. Number of SomaScan protein associations with baseline characteristics: significance, multi-aptamer consistencyin in normalised and non-normalised datasets 16](#_Toc215826035)

[eFigure 1. Study design diagram 17](#_Toc215826036)

[eFigure 2. Cumulative variance explained by principal components for SomaScan protein biomarkers and baseline characteristics 18](#_Toc215826037)

[eFigure 3. Exposure profiles by characteristics type of the top 25 SomaScan protein biomarkers with most positive and negative associations, overall and by sex 19](#_Toc215826038)

[eFigure 4. Associations of selected baseline characteristics with SomaScan protein biomarkers by sex 20](#_Toc215826039)

[eFigure 5. Comparison of associations of selected baseline characteristics and SomaScan protein biomarkers in female and male 21](#_Toc215826040)

[eFigure 6. Age-associated protein biomarkers and their associations with other exposures by sex 22](#_Toc215826041)

[eFigure 7. Post-menopause-associated protein biomarkers and their exposome associations, in females 23](#_Toc215826042)

[eFigure 8. Associations of selected baseline characteristics with protein biomarkers 24](#_Toc215826043)

[eFigure 9. BMI-associated protein biomarkers and their associations with other exposures, in overall analyses 25](#_Toc215826044)

[eFigure 10. BMI-associated protein biomarkers and their associations with other exposures, by sex 26](#_Toc215826045)

[eFigure 11: Exposure profiles of 6597 SomaScan protein biomarkers by abundance level, in normalized dataset 27](#_Toc215826046)

[eFigure 12. Exposure profiles of 6597 SomaScan protein biomarkers overall and by sex, in non-normalized dataset 28](#_Toc215826047)

[eFigure 13. Comparison of associations of selected baseline characteristics and protein biomarkers in normalized and non-normalized datasets 29](#_Toc215826048)

[eFigure 14. Exposure profiles of 6597 SomaScan and 2923 Olink protein biomarkers by platform and normalization status (SomaScan) 30](#_Toc215826049)

[References 31](#_Toc215826050)

#

# Members of the China Kadoorie Biobank Collaborative Group

**International Steering Committee:** Junshi Chen, Zhengming Chen (PI), Robert Clarke, Rory Collins, Liming Li (PI), Jun Lv, Richard Peto, Robin Walters.

**International Co-ordinating Centre, Oxford:** Daniel Avery, Maxim Barnard, Derrick Bennett, Ruth Boxall, Ka Hung Chan, Yiping Chen, Zhengming Chen, Charlotte Clarke, Jonathan Clarke, Robert Clarke, Huaidong Du, Ahmed Edris Mohamed, Hannah Fry, Simon Gilbert, Pek Kei Im, Andri Iona, Maria Kakkoura, Christiana Kartsonaki, Hubert Lam, Kuang Lin, James Liu, Mohsen Mazidi, Iona Millwood, Sam Morris, Qunhua Nie, Alfred Pozarickij, Maryam Rahmati, Paul Ryder, Dan Schmidt, Becky Stevens, Iain Turnbull, Robin Walters, Baihan Wang, Lin Wang, Neil Wright, Ling Yang, Xiaoming Yang, Pang Yao.

**National Co-ordinating Centre, Beijing:** Xiao Han, Can Hou, Qingmei Xia, Chao Liu, Jun Lv, Pei Pei, Dianjanyi Sun, Canqing Yu, Lang Pan

**10 Regional Co-ordinating Centres:**

Qingdao CDC: Zengchang Pang, Ruqin Gao, Shanpeng Li, Haiping Duan, Shaojie Wang, Yongmei Liu, Ranran Du, Yajing Zang, Liang Cheng, Xiaocao Tian, Hua Zhang, Yaoming Zhai, Feng Ning, Xiaohui Sun, Feifei Li. Licang CDC: Silu Lv, Junzheng Wang, Wei Hou. Heilongjiang Provincial CDC: Wei Sun, Shichun Yan, Xiaoming Cui. Nangang CDC: Chi Wang, Zhenyuan Wu,Yanjie Li, Quan Kang. Hainan Provincial CDC: Huiming Luo, Tingting Ou. Meilan CDC: Xiangyang Zheng, Zhendong Guo, Shukuan Wu, Yilei Li, Huimei Li. Jiangsu Provincial CDC: Ming Wu, Yonglin Zhou, Jinyi Zhou, Ran Tao, Jie Yang, Jian Su. Suzhou CDC: Fang Liu, Jun Zhang, Yihe Hu, Yan Lu, Liangcai Ma, Aiyu Tang, Shuo Zhang, Jianrong Jin, Jingchao Liu. Guangxi Provincial CDC: Mei Lin, Zhenzhen Lu. Liuzhou CDC: Lifang Zhou, Changping Xie, Jian Lan,Tingping Zhu,Yun Liu, Liuping Wei, Liyuan Zhou, Ningyu Chen, Yulu Qin, Sisi Wang. Sichuan Provincial CDC: Xianping Wu, Ningmei Zhang, Xiaofang Chen, Xiaoyu Chang. Pengzhou CDC: Mingqiang Yuan, Xia Wu, Xiaofang Chen, Wei Jiang, Jiaqiu Liu, Qiang Sun. Gansu Provincial CDC: Faqing Chen, Xiaolan Ren, Caixia Dong. Maiji CDC: Hui Zhang, Enke Mao, Xiaoping Wang, Tao Wang, Xi zhang. Henan Provincial CDC: Kai Kang, Shixian Feng, Huizi Tian, Lei Fan. Huixian CDC: XiaoLin Li, Huarong Sun, Pan He, Xukui Zhang. Zhejiang Provincial CDC: Min Yu, Ruying Hu, Hao Wang. Tongxiang CDC: Xiaoyi Zhang, Yuan Cao, Kaixu Xie, Lingli Chen, Dun Shen. Hunan Provincial CDC: Xiaojun Li, Donghui Jin, Li Yin, Huilin Liu, Zhongxi Fu. Liuyang CDC: Xin Xu, Hao Zhang, Jianwei Chen,Yuan Peng, Libo Zhang, Chan Qu.

# eTable 1. List of ~7300 SOMAmers targeting human protein biomarkers quantified by the SomaScan Assay v4.1

Saved into the Excel file as supplementary material.

# eTable 2. Baseline characteristics and description of comparison used in analyses

| **Variables** | **Description** |
| --- | --- |
| **Demographics** |  |
| Age | Participant's age at entry into study |
| Sex | Participant's self-reported sex at entry into the study |
| Urban residents | Is the region participant lives in urban compared to rural? |
| Schooling > 9 years | Does participant have greater than 9 years of education (high school; technical school or college; university) compared to less than 9 years of education (middle school; primary school; no formal education) |
| Occupation | Is the participant employed (employed in specific industry; self-employed) compared to unemployed (retired; house wife/ husband; unemployed; other or not stated)? |
| Income ≥ 20,000 yuan/ year | Is the participant's income ≥ 20,000 yuan annually compared to > 20,000 yuan annually? |
| Ownership index (score out of 6) | Derived by aggregating the following binary variables for each participant: possession of health cover; home ownership; access to private sanitation facilities; access to a motor vehicle; access to a phone; engagement in recent leisure travel |
| **Lifestyle factors** |  |
| Current regular alcohol consumption | Does participant currently consume alcohol regularly (weekly) compared to not (never regular; occasional; monthly; ex-regular; reduced intake) |
| Current regular smoker | Is participant currently a regular smoker (smoker) compared to not (never smoker; occasional smoker; ex-regular)? |
| Food diversity score | Food diversity index with a range of 0-24 calculated from a weighted scoring frequency depending on frequency of consumption of following food groups: dairy; eggs; fish; fresh fruit; fresh vegetables; red meat; wholegrains; poultry; preserved vegetables; rice; soybean; wheat. |
| Rapeseed vs. other plant (excluding lard) | Does participant use rapeseed oil to cook compared to other plant oils (peanut; soybean; other), excluding lard, or not? |
| Total physical activity | MET-hours per day engaging in physical activity |
| **Environmental** |  |
| Outdoor temperature | Mean daily temperature in region (°C) |
| Heating fuel | Does participant currently use clean heating fuel (gas; electric; central heating) or not (coal; wood; other)? |
| Cooking fuel | Does participant currently use clean cooking fuel (gas or electric) or not (coal; wood; other)? |
| **Health and wellbeing** |  |
| Self-rated health | Does participant have poor self-rated health compared to not poor self-rated health (fair; good; excellent)? |
| Respiratory disease | Has participant self-reported as being doctor diagnosed with any of the following: asthma; COPD; TB; emphysema or bronchitis? |
| Kidney/ liver disease | Has participant self-reported as being doctor diagnosed with any of the following: kidney disease; cirrhosis or hepatitis? |
| HBsAg+ | Has participant tested positive for the presence of HBsAg? (those with 'unclear' result set to missing) |
| Diabetes | Does participant have diabetes or not? This is based on a combination of self-reported doctor diagnosed cases and screen-detected from an RPG test, where an RPG level of ≥ 11.1 mmol/L resulted in a diabetes diagnosis, or if fasting blood glucose was ≥ 7 mmol/L. |
| Cancer | Has participant been diagnosed with cancer by a doctor or not? |
| Life satisfaction | Is participant unsatisfied with their life generally (very unsatisfied; unsatisfied) compared to not being unsatisfied with their life generally (neither satisfied nor unsatisfied; satisfied; very satisfied)? |
| Mental disorder | Has participant been diagnosed with a mental health disorder (major depression; generalised anxiety; other psychiatric disorder)? |
| **Clinical measurements** |  |
| BMI | BMI of participant (kg/ m²), measured at baseline |
| Standing height | Standing height of participant in cm, measured at baseline |
| SBP | Participant's mean systolic blood pressure in mmHg |
| DBP | Participant's mean diastolic blood pressure in mmHg |
| Heart rate | Participant's heart rate in bpm |
| Max CO ppm measurement | Participant's maximum CO measurement |
| FEV1/ FVC ratio | Participant's FEV1/ FVC ratio |
| RBG | Participant's random plasma glucose in mmol/L |
| Fasting time | Participant's fasting time: hours since they last ate |
| **Reproductive factors** |  |
| Age at menarche | Among females, age at which participant had their first period |
| Age at menopause | Among females, age at which menopause began |
| Post-menopausal | Among females, has participant gone through menopause (compared to not menopausal; currently going through menopause) |
| Parity | Among females, has participant ever given birth (live or stillbirth) |
| Age at first live birth | Age at first live birth |
| **Derived indices** |  |
| Healthy lifestyle index | A derived score comprised of baseline variables related to smoking status, alcohol consumption, physical activity, diet and body shape, indicating a healthy lifestyle that were measured by use of self-reported questionnaires, or physical examinations.^1, 2^ |
| Frailty index | A proxy measure of accelerated biological aging, comprised of 28 baseline variables indicative of health status deficits that were measured by use of self-reported questionnaires, or physical examinations.^3^ |
| All binary variables in the table are described as yes vs. no (description on left vs. description on right) where 1 = yes; 0 = no. Daily mean weather measurements taken on the same day participant attended assessment centre. BMI: Body mass index; CO: Carbon monoxide; COPD: Chronic obstructive pulmonary disease; DBP: Diastolic blood pressure; FEV1/FVC: Forced expiratory volume in 1 second/ forced vital capacity; HBsAg: hepatitis B surface antigen; MET: Metabolic equivalent task; RPG: Random plasma glucose; SBP: Systolic blood pressure; TB: Tuberculosis | |

# eTable 3. Principal component analysis for SomaScan protein biomarkers and baseline characteristics

Saved into the Excel file as supplementary material.

# eTable 4. Effect estimates from the association of baseline characteristics with proteins

Saved into the Excel file as supplementary material.

# eTable 5. Number of proteins significantly associated with baseline characteristic after sequential adjustments, in overall analyses

| **Characteristics** | **Adjustments** | | | | | |
| --- | --- | --- | --- | --- | --- | --- |
|  | **Basic ᵃ** | **+ fasting time ᵇ** | **+ outdoor temperature ᶜ** | **+ BMI ᵈ** | **+ SBP ᵉ** | **+ mutual ᶠ** |
| **Demographics** |  |  |  |  |  |  |
| Age, years | 993 | 989 | 982 | 989 | 905 | 658 |
| Sex | 989 | 997 | 996 | 1027 | 1013 | 593 |
| Urban residents | 883 | 873 | 858 | 729 | 726 | 227 |
| Schooling | 0 | 0 | 0 | 1 | 1 | 0 |
| Employed | 4 | 3 | 1 | 1 | 1 | 0 |
| Household income | 4 | 5 | 7 | 1 | 1 | 0 |
| Ownership index ᵍ | 13 | 11 | 13 | 1 | 1 | 0 |
| **Lifestyle** |  |  |  |  |  |  |
| Regular alcohol drinker | 106 | 106 | 99 | 102 | 101 | 89 |
| Current smoker | 38 | 35 | 36 | 49 | 51 | 18 |
| Diet |  |  |  |  |  |  |
| Food diversity score ʰ | 3 | 3 | 3 | 1 | 1 | 0 |
| Rapeseed oil | 7 | 6 | 7 | 5 | 5 | 2 |
| Physical activity | 9 | 9 | 9 | 10 | 10 | 1 |
| **Environmental** |  |  |  |  |  |  |
| Outdoor temperature | 807 | 802 | 802 | 810 | 750 | 751 |
| Clean heating fuel | 0 | 0 | 0 | 0 | 0 | 0 |
| Clean cooking fuel | 1 | 1 | 0 | 0 | 0 | 0 |
| **Health and wellbeing** |  |  |  |  |  |  |
| Self-rated health | 0 | 0 | 1 | 0 | 0 | 0 |
| Respiratory disease | 1 | 1 | 1 | 0 | 0 | 0 |
| Kidney/liver disease | 5 | 5 | 5 | 5 | 6 | 5 |
| HBsAg+ | 452 | 455 | 468 | 483 | 481 | 522 |
| Diabetes | 263 | 262 | 257 | 189 | 166 | 168 |
| Cancer | 9 | 9 | 10 | 10 | 11 | 11 |
| Life satisfaction | 0 | 0 | 0 | 0 | 0 | 0 |
| Mental disorder | 3 | 3 | 3 | 3 | 3 | 3 |
| **Clinical measurements** |  |  |  |  |  |  |
| BMI | 1030 | 1032 | 1035 | 1035 | 860 | 838 |
| Standing height | 20 | 20 | 22 | 22 | 23 | 18 |
| SBP | 504 | 505 | 293 | 53 | 53 | 38 |
| DBP | 259 | 253 | 233 | 50 | 50 | 35 |
| Heart rate | 182 | 185 | 181 | 159 | 159 | 146 |
| Exhaled CO | 13 | 13 | 12 | 14 | 14 | 4 |
| FEV1/FVC ratio | 3 | 4 | 2 | 0 | 0 | 0 |
| RPG | 322 | 331 | 343 | 278 | 255 | 58 |
| Fasting time | 85 | 85 | 86 | 89 | 88 | 89 |
| **Reproductive factors** |  |  |  |  |  |  |
| Age at menarche | 0 | 0 | 0 | 0 | 0 | 0 |
| Age at menopause | 0 | 0 | 0 | 0 | 0 | 0 |
| Post-menopausal | 0 | 0 | 0 | 0 | 0 | 0 |
| Parity | 0 | 0 | 0 | 0 | 0 | 0 |
| Age at first live birth | 0 | 0 | 0 | 0 | 0 | 0 |
| **Lifestyle index ⁱ** | 313 | 314 | 307 | 307 | 269 | 204 |
| **Frailty index ʲ** | 475 | 475 | 465 | 465 | 465 | 440 |
| ᵃ Analyses are adjusted for age, age²,sex, study area, and plate ID, where appropriate. Analyses performed based on ANML data. Bonferroni (PCA) corrected p-value < 0.05 | | | | | | |
| ᵇ fasting time and fasting time² | | | | | | |
| ᶜ Analyses are additionally adjusted for outdoor temperature and outdoor temperature^2^, where appropriate. | | | | | | |
| ᵈ Analyses are additionally adjusted for BMI, where appropriate. | | | | | | |
| ᵉ Analyses are additionally adjusted for SBP, where appropriate. | | | | | | |
| ᶠ Analyses are additionally adjusted for education, employment, income, ownership index, alcohol, smoking, food diversity score, physical activity, self-rated health, diabetes, life satisfaction, mental disorder, exhaled CO, | | | | | | |
| where appropriate. | | | | | | |
| ᵍ 6-point index of qualitative measures of living standards | | | | | | |
| ʰ 24-point index of frequency of intake in 12 food groups | | | | | | |
| ⁱ 5-point index of low-risk lifestyle characteristics | | | | | | |
| ʲ 28-point index of accumulation of health deficits and physical activity | | | | | | |
| Abbreviations: ANML: Adaptive normalisation by maximum likelihood; BMI: Body mass index; CO: carbon-monoxide; DBP: Diastolic blood pressure; HBsAg+: Hepatitis B virus surface antigen seropositive; RPG: Random Plasma Glucose | | | | | | |

# eTable 6. Number of SomaScan proteins significantly associated with only one baseline characteristic, overall and by sex, in normalized and non-normalised datasets

| **Characteristics** | **Normalised** | | |  | **Non-normalised** | | |
| --- | --- | --- | --- | --- | --- | --- | --- |
|  | Female | Male | All |  | Female | Male | All |
| **Demographics** |  |  |  |  |  |  |  |
| Age | 269 | 239 | 191 |  | 498 | 241 | 183 |
| Sex | - | - | 180 |  | - | - | 96 |
| Urban residents | 171 | 135 | 135 |  | 133 | 97 | 106 |
| Schooling | 0 | 0 | 0 |  | 0 | 0 | 0 |
| Employed | 0 | 0 | 0 |  | 0 | 0 | 0 |
| Income | 0 | 1 | 0 |  | 0 | 0 | 0 |
| Ownership index | 0 | 0 | 0 |  | 0 | 1 | 0 |
| **Lifestyle habits** |  |  |  |  |  |  |  |
| Regular alcohol drinker | 2 | 24 | 4 |  | 0 | 15 | 2 |
| Current smoker | 4 | 4 | 0 |  | 3 | 8 | 0 |
| Diet |  |  |  |  |  |  |  |
| Food diversity score | 0 | 0 | 0 |  | 0 | 0 | 0 |
| Rapeseed oil | 0 | 0 | 0 |  | 0 | 0 | 0 |
| Physical activity | 0 | 0 | 0 |  | 0 | 0 | 0 |
| **Environmental** |  |  |  |  |  |  |  |
| Outdoor temperature | 379 | 266 | 354 |  | 214 | 184 | 104 |
| Clean heating fuel | 0 | 0 | 0 |  | 0 | 0 | 0 |
| Clean cooking fuel | 0 | 0 | 0 |  | 0 | 0 | 0 |
| **Health and wellbeing** |  |  |  |  |  |  |  |
| Prior physical health status |  |  |  |  |  |  |  |
| Self-rated health | 0 | 0 | 0 |  | 0 | 1 | 0 |
| Respiratory disease | 0 | 0 | 1 |  | 0 | 0 | 0 |
| Kidney/liver disease | 5 | 9 | 2 |  | 2 | 28 | 4 |
| HBsAg+ | 74 | 62 | 107 |  | 62 | 27 | 43 |
| Diabetes | 2 | 2 | 1 |  | 1 | 3 | 2 |
| Cancer | 13 | 21 | 8 |  | 11 | 10 | 1 |
| Mental wellbeing |  |  |  |  |  |  |  |
| Life satisfaction | 1 | 1 | 0 |  | 0 | 1 | 0 |
| Mental disorder | 6 | 7 | 2 |  | 4 | 4 | 0 |
| **Clinical measurements** |  |  |  |  |  |  |  |
| BMI | 130 | 124 | 118 |  | 285 | 671 | 770 |
| Height | 0 | 0 | 0 |  | 0 | 2 | 0 |
| SBP | 9 | 0 | 5 |  | 12 | 0 | 2 |
| DBP | 0 | 1 | 2 |  | 0 | 7 | 2 |
| Heart rate | 1 | 5 | 0 |  | 6 | 7 | 12 |
| Exhaled CO | 1 | 2 | 0 |  | 0 | 3 | 0 |
| FEV1/FVC ratio | 0 | 0 | 0 |  | 0 | 0 | 0 |
| RPG | 9 | 17 | 5 |  | 18 | 27 | 10 |
| Fasting time | 10 | 12 | 10 |  | 2 | 12 | 4 |
| **Reproductive factors** |  |  |  |  |  |  |  |
| Age at menarche | 0 | - | 0 |  | 0 | - | 0 |
| Age at menopause | 1 | - | 0 |  | 0 | - | 0 |
| Post-menopausal | 2 | - | 0 |  | 2 | - | 2 |
| Parity | 28 | - | 27 |  | 24 | - | 14 |
| Age at first live birth | 0 | - | 0 |  | 0 | - | 0 |
| **Composite scores** |  |  |  |  |  |  |  |
| Lifestyle index | 0 | 9 | 1 |  | 0 | 15 | 2 |
| Frailty index | 5 | 1 | 2 |  | 13 | 28 | 23 |

# eTable 7. Number of proteins significantly associated with baseline characteristic after sequential adjustments, in females

| **Characteristics** | **Adjustments** | | | | | |
| --- | --- | --- | --- | --- | --- | --- |
|  | **Basic ᵃ** | **+ fasting time ᵇ** | **+ outdoor temperature ᶜ** | **+ BMI ᵈ** | **+ SBP ᵉ** | **+ mutual ᶠ** |
| **Demographics** |  |  |  |  |  |  |
| Age, years | 754 | 739 | 735 | 727 | 609 | 477 |
| Urban residents | 498 | 490 | 506 | 457 | 444 | 124 |
| Schooling | 0 | 0 | 0 | 1 | 1 | 0 |
| Employed | 0 | 0 | 0 | 0 | 0 | 0 |
| Household income | 0 | 0 | 0 | 0 | 0 | 0 |
| Ownership index ᵍ | 0 | 0 | 0 | 0 | 0 | 0 |
| **Lifestyle** |  |  |  |  |  |  |
| Regular alcohol drinker | 2 | 2 | 2 | 2 | 2 | 2 |
| Current smoker | 5 | 5 | 5 | 5 | 5 | 3 |
| Diet |  |  |  |  |  |  |
| Food diversity score ʰ | 0 | 0 | 0 | 0 | 0 | 0 |
| Rapeseed oil | 3 | 3 | 5 | 4 | 4 | 0 |
| Physical activity | 0 | 0 | 0 | 0 | 0 | 0 |
| **Environmental** |  |  |  |  |  |  |
| Outdoor temperature | 578 | 567 | 567 | 581 | 498 | 497 |
| Clean heating fuel | 0 | 0 | 0 | 0 | 0 | 0 |
| Clean cooking fuel | 2 | 1 | 3 | 1 | 1 | 0 |
| **Health and wellbeing** |  |  |  |  |  |  |
| Self-rated health | 0 | 0 | 0 | 0 | 0 | 0 |
| Respiratory disease | 0 | 0 | 0 | 0 | 0 | 0 |
| Kidney/liver disease | 5 | 5 | 6 | 5 | 5 | 4 |
| HBsAg+ | 255 | 258 | 260 | 265 | 266 | 269 |
| Diabetes | 166 | 162 | 166 | 127 | 94 | 99 |
| Cancer | 14 | 14 | 14 | 14 | 14 | 14 |
| Life satisfaction | 1 | 1 | 1 | 1 | 1 | 0 |
| Mental disorder | 7 | 7 | 7 | 7 | 7 | 8 |
| **Clinical measurements** |  |  |  |  |  |  |
| BMI | 592 | 602 | 595 | 595 | 478 | 452 |
| Standing height | 3 | 3 | 3 | 3 | 3 | 2 |
| SBP | 273 | 269 | 136 | 23 | 23 | 21 |
| DBP | 71 | 67 | 60 | 11 | 11 | 10 |
| Heart rate | 63 | 62 | 61 | 55 | 55 | 47 |
| Exhaled CO | 1 | 1 | 1 | 1 | 1 | 2 |
| FEV1/FVC ratio | 0 | 0 | 0 | 0 | 0 | 0 |
| RPG | 252 | 248 | 251 | 180 | 161 | 21 |
| Fasting time | 45 | 45 | 45 | 50 | 48 | 47 |
| **Reproductive factors** |  |  |  |  |  |  |
| Age at menarche | 0 | 0 | 0 | 0 | 0 | 0 |
| Age at menopause | 0 | 0 | 1 | 1 | 1 | 1 |
| Post-menopausal | 61 | 59 | 58 | 59 | 59 | 56 |
| Parity | 30 | 29 | 29 | 29 | 29 | 28 |
| Age at first live birth | 0 | 0 | 0 | 0 | 0 | 0 |
| **Lifestyle index ⁱ** | 111 | 112 | 106 | 106 | 90 | 70 |
| **Frailty index ʲ** | 220 | 222 | 226 | 226 | 226 | 208 |
| ᵃ Analyses are adjusted for age, age², study area, and plate ID, where appropriate. Analyses performed based on ANML data.Bonferroni (PCA) corrected p-value < 0.05 | | | | | | |
| ᵇ fasting time and fasting time² | | | | | | |
| ᶜ Analyses are additionally adjusted for outdoor temperature and outdoor temperature^2^, where appropriate. | | | | | | |
| ᵈ Analyses are additionally adjusted for BMI, where appropriate. | | | | | | |
| ᵉ Analyses are additionally adjusted for SBP, where appropriate. | | | | | | |
| ᶠ Analyses are additionally adjusted for education, employment, income, ownership index, alcohol, smoking, food diversity score, physical activity, self-rated health, diabetes, life satisfaction, mental disorder, exhaled CO, | | | | | | |
| where appropriate. | | | | | | |
| ᵍ 6-point index of qualitative measures of living standards | | | | | | |
| ʰ 24-point index of frequency of intake in 12 food groups | | | | | | |
| ⁱ 5-point index of low-risk lifestyle characteristics | | | | | | |
| ʲ 28-point index of accumulation of health deficits and physical activity | | | | | | |
| Abbreviations: ANML: Adaptive normalisation by maximum likelihood; BMI: Body mass index; CO: carbon-monoxide; DBP: Diastolic blood pressure; HBsAg+: Hepatitis B virus surface antigen seropositive; RPG: Random Plasma Glucose | | | | | | |

# eTable 8. Number of proteins significantly associated with baseline characteristic after sequential adjustments, in males

| **Characteristics** | **Adjustments** | | | | | |
| --- | --- | --- | --- | --- | --- | --- |
|  | **Basic ᵃ** | **+ fasting time ᵇ** | **+ outdoor temperature ᶜ** | **+ BMI ᵈ** | **+ SBP ᵉ** | **+ mutual ᶠ** |
| **Demographics** |  |  |  |  |  |  |
| Age, years | 589 | 588 | 593 | 546 | 551 | 312 |
| Urban residents | 456 | 452 | 425 | 197 | 205 | 89 |
| Schooling | 0 | 0 | 0 | 0 | 0 | 0 |
| Employed | 0 | 0 | 0 | 0 | 0 | 0 |
| Household income | 5 | 6 | 5 | 3 | 4 | 0 |
| Ownership index ᵍ | 15 | 15 | 16 | 3 | 3 | 0 |
| **Lifestyle** |  |  |  |  |  |  |
| Regular alcohol drinker | 86 | 86 | 85 | 86 | 80 | 70 |
| Current smoker | 25 | 25 | 24 | 27 | 27 | 7 |
| Diet |  |  |  |  |  |  |
| Food diversity score ʰ | 0 | 0 | 0 | 0 | 0 | 0 |
| Rapeseed oil | 0 | 0 | 0 | 0 | 0 | 0 |
| Physical activity | 3 | 3 | 3 | 1 | 1 | 0 |
| **Environmental** |  |  |  |  |  |  |
| Outdoor temperature | 339 | 336 | 336 | 339 | 309 | 311 |
| Clean heating fuel | 0 | 0 | 0 | 0 | 0 | 0 |
| Clean cooking fuel | 0 | 0 | 0 | 0 | 0 | 0 |
| **Health and wellbeing** |  |  |  |  |  |  |
| Self-rated health | 0 | 0 | 0 | 0 | 0 | 1 |
| Respiratory disease | 0 | 0 | 0 | 0 | 0 | 0 |
| Kidney/liver disease | 16 | 16 | 19 | 19 | 20 | 18 |
| HBsAg+ | 115 | 115 | 115 | 121 | 119 | 118 |
| Diabetes | 36 | 36 | 34 | 36 | 37 | 31 |
| Cancer | 28 | 28 | 27 | 26 | 23 | 24 |
| Life satisfaction | 1 | 1 | 1 | 2 | 2 | 2 |
| Mental disorder | 7 | 8 | 9 | 9 | 9 | 10 |
| **Clinical measurements** |  |  |  |  |  |  |
| BMI | 497 | 500 | 498 | 498 | 426 | 379 |
| Standing height | 5 | 5 | 6 | 6 | 9 | 4 |
| SBP | 81 | 79 | 87 | 8 | 8 | 5 |
| DBP | 85 | 82 | 74 | 16 | 16 | 8 |
| Heart rate | 49 | 44 | 40 | 42 | 42 | 36 |
| Exhaled CO | 19 | 19 | 20 | 22 | 22 | 5 |
| FEV1/FVC ratio | 4 | 4 | 4 | 2 | 2 | 1 |
| RPG | 76 | 73 | 77 | 79 | 79 | 20 |
| Fasting time | 20 | 20 | 21 | 23 | 23 | 24 |
| **Lifestyle index ⁱ** | 95 | 96 | 96 | 96 | 66 | 43 |
| **Frailty index ʲ** | 78 | 80 | 82 | 82 | 82 | 87 |
| ᵃ Analyses are adjusted for age, age², study area, and plate ID, where appropriate. Analyses performed based on ANML data.Bonferroni (PCA) corrected p-value < 0.05 | | | | | | |
| ᵇ fasting time and fasting time² | | | | | | |
| ᶜ Analyses are additionally adjusted for outdoor temperature and outdoor temperature^2^, where appropriate. | | | | | | |
| ᵈ Analyses are additionally adjusted for BMI, where appropriate. | | | | | | |
| ᵉ Analyses are additionally adjusted for SBP, where appropriate. | | | | | | |
| ᶠ Analyses are additionally adjusted for education, employment, income, ownership index, alcohol, smoking, food diversity score, physical activity, self-rated health, diabetes, life satisfaction, mental disorder, exhaled CO, | | | | | | |
| where appropriate. | | | | | | |
| ᵍ 6-point index of qualitative measures of living standards | | | | | | |
| ʰ 24-point index of frequency of intake in 12 food groups | | | | | | |
| ⁱ 5-point index of low-risk lifestyle characteristics | | | | | | |
| ʲ 28-point index of accumulation of health deficits and physical activity | | | | | | |
| Abbreviations: ANML: Adaptive normalisation by maximum likelihood; BMI: Body mass index; CO: carbon-monoxide; DBP: Diastolic blood pressure; HBsAg+: Hepatitis B virus surface antigen seropositive; RPG: Random Plasma Glucose | | | | | | |

# eTable 9. Number of SomaScan proteins significantly associated with baseline characteristics by abundance level

| **Characteristics** | **Abundance level** | | | **All** |
| --- | --- | --- | --- | --- |
|  | Low | Moderate | High |  |
|  | (n=5,562) | (n=1,034) | (n=173) | (n=6,597) |
| **Demographics** |  |  |  |  |
| Age | 579 (10.4) | 332 (32.1) | 71 (41.0) | 982 (14.9) |
| Sex | 610 (11.0) | 319 (30.9) | 67 (38.7) | 996 (15.1) |
| Urban residents | 446 ( 8.0) | 340 (32.9) | 72 (41.6) | 858 (13.0) |
| Schooling | 0 ( 0.0) | 0 ( 0.0) | 0 ( 0.0) | 0 ( 0.0) |
| Employed | 0 ( 0.0) | 1 ( 0.1) | 0 ( 0.0) | 1 ( 0.0) |
| Income | 0 ( 0.0) | 6 ( 0.6) | 1 ( 0.6) | 7 ( 0.1) |
| Ownership index | 5 ( 0.1) | 7 ( 0.7) | 1 ( 0.6) | 13 ( 0.2) |
| **Lifestyle habits** |  |  |  |  |
| Regular alcohol drinker | 39 ( 0.7) | 37 ( 3.6) | 23 (13.3) | 99 ( 1.5) |
| Current smoker | 23 ( 0.4) | 10 ( 1.0) | 3 ( 1.7) | 36 ( 0.5) |
| Diet |  |  |  |  |
| Food diversity score | 2 ( 0.0) | 0 ( 0.0) | 1 ( 0.6) | 3 ( 0.0) |
| Rapeseed oil | 0 ( 0.0) | 6 ( 0.6) | 1 ( 0.6) | 7 ( 0.1) |
| Physical activity | 5 ( 0.1) | 4 ( 0.4) | 0 ( 0.0) | 9 ( 0.1) |
| **Environmental** |  |  |  |  |
| Outdoor temperature | 582 (10.5) | 202 (19.5) | 18 (10.4) | 802 (12.2) |
| Clean heating fuel | 0 ( 0.0) | 0 ( 0.0) | 0 ( 0.0) | 0 ( 0.0) |
| Clean cooking fuel | 0 ( 0.0) | 0 ( 0.0) | 0 ( 0.0) | 0 ( 0.0) |
| **Health and wellbeing** |  |  |  |  |
| Prior physical health status |  |  |  |  |
| Self-rated health | 1 ( 0.0) | 0 ( 0.0) | 0 ( 0.0) | 1 ( 0.0) |
| Respiratory disease | 1 ( 0.0) | 0 ( 0.0) | 0 ( 0.0) | 1 ( 0.0) |
| Kidney/liver disease | 3 ( 0.1) | 1 ( 0.1) | 1 ( 0.6) | 5 ( 0.1) |
| HBsAg+ | 271 ( 4.9) | 157 (15.2) | 40 (23.1) | 468 ( 7.1) |
| Diabetes | 112 ( 2.0) | 120 (11.6) | 25 (14.5) | 257 ( 3.9) |
| Cancer | 10 ( 0.2) | 0 ( 0.0) | 0 ( 0.0) | 10 ( 0.2) |
| Mental wellbeing |  |  |  |  |
| Life satisfaction | 0 ( 0.0) | 0 ( 0.0) | 0 ( 0.0) | 0 ( 0.0) |
| Mental disorder | 3 ( 0.1) | 0 ( 0.0) | 0 ( 0.0) | 3 ( 0.0) |
| **Clinical measurements** |  |  |  |  |
| BMI | 590 (10.6) | 372 (36.0) | 73 (42.2) | 1035 (15.7) |
| Height | 14 ( 0.3) | 8 ( 0.8) | 0 ( 0.0) | 22 ( 0.3) |
| SBP | 132 ( 2.4) | 116 (11.2) | 45 (26.0) | 293 ( 4.4) |
| DBP | 90 ( 1.6) | 98 ( 9.5) | 45 (26.0) | 233 ( 3.5) |
| Heart rate | 72 ( 1.3) | 92 ( 8.9) | 17 ( 9.8) | 181 ( 2.7) |
| Exhaled CO | 8 ( 0.1) | 3 ( 0.3) | 1 ( 0.6) | 12 ( 0.2) |
| FEV1/FVC ratio | 0 ( 0.0) | 1 ( 0.1) | 1 ( 0.6) | 2 ( 0.0) |
| RPG | 172 ( 3.1) | 146 (14.1) | 25 (14.5) | 343 ( 5.2) |
| Fasting time | 60 ( 1.1) | 24 ( 2.3) | 2 ( 1.2) | 86 ( 1.3) |
| **Reproductive factors** |  |  |  |  |
| Age at menarche | 0 ( 0.0) | 0 ( 0.0) | 0 ( 0.0) | 0 ( 0.0) |
| Age at menopause | 1 ( 0.0) | 0 ( 0.0) | 0 ( 0.0) | 1 ( 0.0) |
| Post-menopausal | 26 ( 0.5) | 28 ( 2.7) | 4 ( 2.3) | 58 ( 0.9) |
| Parity | 27 ( 0.5) | 2 ( 0.2) | 0 ( 0.0) | 29 ( 0.4) |
| Age at first live birth | 0 ( 0.0) | 0 ( 0.0) | 0 ( 0.0) | 0 ( 0.0) |
| **Composite scores** |  |  |  |  |
| Lifestyle index | 142 ( 2.6) | 131 (12.7) | 34 (19.7) | 307 ( 4.7) |
| Frailty index | 231 ( 4.2) | 186 (18.0) | 48 (27.7) | 465 ( 7.0) |

# eTable 10. Number of SomaScan proteins significantly associated with baseline characteristics, in normalised and non-normalised datasets

| **Characteristics** | **Normalised** | | |  | **Non-normalised** | | |
| --- | --- | --- | --- | --- | --- | --- | --- |
|  | Female | Male | All |  | Female | Male | All |
| **Demographics** |  |  |  |  |  |  |  |
| Age | 735 | 593 | 982 |  | 1511 | 528 | 1363 |
| Sex | - | - | 996 |  | - | - | 902 |
| Urban residents | 506 | 425 | 858 |  | 496 | 358 | 968 |
| Schooling | 0 | 0 | 0 |  | 1 | 0 | 5 |
| Employed | 0 | 0 | 1 |  | 3 | 0 | 2 |
| Income | 0 | 5 | 7 |  | 0 | 7 | 2 |
| Ownership index | 0 | 16 | 13 |  | 0 | 13 | 10 |
| **Lifestyle habits** |  |  |  |  |  |  |  |
| Regular alcohol drinker | 2 | 85 | 99 |  | 0 | 67 | 77 |
| Current smoker | 5 | 24 | 36 |  | 5 | 24 | 37 |
| Diet |  |  |  |  |  |  |  |
| Food diversity score | 0 | 0 | 3 |  | 0 | 1 | 1 |
| Rapeseed oil | 5 | 0 | 7 |  | 2 | 0 | 3 |
| Physical activity | 0 | 3 | 9 |  | 0 | 1 | 4 |
| **Environmental** |  |  |  |  |  |  |  |
| Outdoor temperature | 567 | 336 | 802 |  | 464 | 259 | 678 |
| Clean heating fuel | 0 | 0 | 0 |  | 0 | 0 | 0 |
| Clean cooking fuel | 3 | 0 | 0 |  | 0 | 0 | 0 |
| **Health and wellbeing** |  |  |  |  |  |  |  |
| Prior physical health status |  |  |  |  |  |  |  |
| Self-rated health | 0 | 0 | 1 |  | 0 | 1 | 1 |
| Respiratory disease | 0 | 0 | 1 |  | 0 | 0 | 0 |
| Kidney/liver disease | 6 | 19 | 5 |  | 3 | 49 | 7 |
| HBsAg+ | 260 | 115 | 468 |  | 247 | 77 | 396 |
| Diabetes | 166 | 34 | 257 |  | 278 | 50 | 482 |
| Cancer | 14 | 27 | 10 |  | 18 | 14 | 8 |
| Mental wellbeing |  |  |  |  |  |  |  |
| Life satisfaction | 1 | 1 | 0 |  | 0 | 1 | 0 |
| Mental disorder | 7 | 9 | 3 |  | 5 | 7 | 0 |
| **Clinical measurements** |  |  |  |  |  |  |  |
| BMI | 595 | 498 | 1035 |  | 1254 | 1152 | 2786 |
| Height | 3 | 6 | 22 |  | 2 | 6 | 19 |
| SBP | 136 | 87 | 293 |  | 467 | 136 | 907 |
| DBP | 60 | 74 | 233 |  | 212 | 168 | 674 |
| Heart rate | 61 | 40 | 181 |  | 176 | 70 | 662 |
| Exhaled CO | 1 | 20 | 12 |  | 0 | 18 | 12 |
| FEV1/FVC ratio | 0 | 4 | 2 |  | 0 | 3 | 1 |
| RPG | 251 | 77 | 343 |  | 444 | 122 | 651 |
| Fasting time | 45 | 21 | 86 |  | 39 | 21 | 74 |
| **Reproductive factors** |  |  |  |  |  |  |  |
| Age at menarche | 0 | - | 0 |  | 0 | - | 0 |
| Age at menopause | 1 | - | 1 |  | 0 | - | 0 |
| Post-menopausal | 58 | - | 58 |  | 198 | - | 196 |
| Parity | 29 | - | 29 |  | 30 | - | 30 |
| Age at first live birth | 0 | - | 0 |  | 0 | - | 0 |
| **Composite scores** |  |  |  |  |  |  |  |
| Lifestyle index | 106 | 96 | 307 |  | 213 | 180 | 778 |
| Frailty index | 226 | 82 | 465 |  | 545 | 306 | 1478 |

# eTable 11. Number of SomaScan protein associations with baseline characteristics: significance, multi-aptamer consistencyin in normalised and non-normalised datasets

| **Characteristics** | **Normalised** | | |  | **Non-normalised** | | |
| --- | --- | --- | --- | --- | --- | --- | --- |
|  | **Significant proteins*** | **Proteins with multiple aptamers**** | **Directional consistency^†^** |  | **Significant proteins*** | **Proteins with multiple aptamers**** | **Directional consistency^†^** |
| **Demographics** |  |  |  |  |  |  |  |
| Age | 982 | 136 | 116 |  | 1363 | 206 | 202 |
| Sex | 996 | 121 | 101 |  | 902 | 120 | 101 |
| Urban residents | 858 | 127 | 110 |  | 968 | 139 | 121 |
| Schooling | 0 | 0 | 0 |  | 5 | 1 | 1 |
| Employed | 1 | 0 | 0 |  | 2 | 0 | 0 |
| Income | 7 | 2 | 2 |  | 2 | 2 | 2 |
| Ownership index | 13 | 4 | 4 |  | 10 | 4 | 4 |
| **Lifestyle habits** |  |  |  |  |  |  |  |
| Regular alcohol drinker | 99 | 12 | 12 |  | 77 | 9 | 9 |
| Current smoker | 36 | 10 | 10 |  | 37 | 12 | 12 |
| Diet |  |  |  |  |  |  |  |
| Food diversity score | 3 | 1 | 1 |  | 1 | 1 | 1 |
| Rapeseed oil | 7 | 3 | 3 |  | 3 | 2 | 2 |
| Physical activity | 9 | 0 | 0 |  | 4 | 0 | 0 |
| **Environmental** |  |  |  |  |  |  |  |
| Outdoor temperature | 802 | 71 | 61 |  | 678 | 58 | 50 |
| Clean heating fuel | 0 | 0 | 0 |  | 0 | 0 | 0 |
| Clean cooking fuel | 0 | 0 | 0 |  | 0 | 0 | 0 |
| **Health and wellbeing** |  |  |  |  |  |  |  |
| Prior physical health status |  |  |  |  |  |  |  |
| Self-rated health | 1 | 0 | 1 |  | 1 | 0 | 0 |
| Respiratory disease | 1 | 0 | 1 |  | 0 | 0 | 0 |
| Kidney/liver disease | 5 | 0 | 0 |  | 7 | 0 | 0 |
| HBsAg+ | 468 | 56 | 45 |  | 396 | 49 | 40 |
| Diabetes | 257 | 43 | 40 |  | 482 | 73 | 72 |
| Cancer | 10 | 0 | 0 |  | 8 | 0 | 0 |
| Mental wellbeing |  |  |  |  |  |  |  |
| Life satisfaction | 0 | 0 | 0 |  | 0 | 0 | 0 |
| Mental disorder | 3 | 0 | 0 |  | 0 | 0 | 0 |
| **Clinical measurements** |  |  |  |  |  |  |  |
| BMI | 1035 | 137 | 119 |  | 2786 | 304 | 287 |
| Height | 22 | 4 | 4 |  | 19 | 4 | 4 |
| SBP | 293 | 48 | 43 |  | 907 | 118 | 118 |
| DBP | 233 | 47 | 42 |  | 674 | 84 | 84 |
| Heart rate | 181 | 46 | 42 |  | 662 | 90 | 90 |
| Exhaled CO | 12 | 3 | 3 |  | 12 | 3 | 3 |
| FEV1/FVC ratio | 2 | 1 | 1 |  | 1 | 1 | 1 |
| RPG | 343 | 52 | 50 |  | 651 | 98 | 96 |
| Fasting time | 86 | 13 | 12 |  | 74 | 13 | 13 |
| **Reproductive factors** |  |  |  |  |  |  |  |
| Age at menarche | 0 | - | 0 |  | 0 | 0 | 0 |
| Age at menopause | 1 | - | 1 |  | 0 | 0 | 0 |
| Post-menopausal | 58 | - | 58 |  | 196 | 34 | 34 |
| Parity | 29 | - | 29 |  | 30 | 1 | 1 |
| Age at first live birth | 0 | - | 0 |  | 0 | 0 | 0 |
| **Composite scores** |  |  |  |  |  |  |  |
| Lifestyle index | 307 | 51 | 44 |  | 778 | 96 | 93 |
| Frailty index | 465 | 62 | 55 |  | 1478 | 183 | 180 |

Analyses are adjusted for age, age², study area, and plate ID, fasting time and fasting time², outdoor temperature and outdoor temperature^2^, where appropriate.

* Bonferroni (PCA) corrected p-value < 0.05.

** Among significant proteins with >1 aptamer, number nominally significant (p < 0.05).
† Number of proteins whose aptamers showed consistent direction of effect.

# eFigure 1. Study design diagram


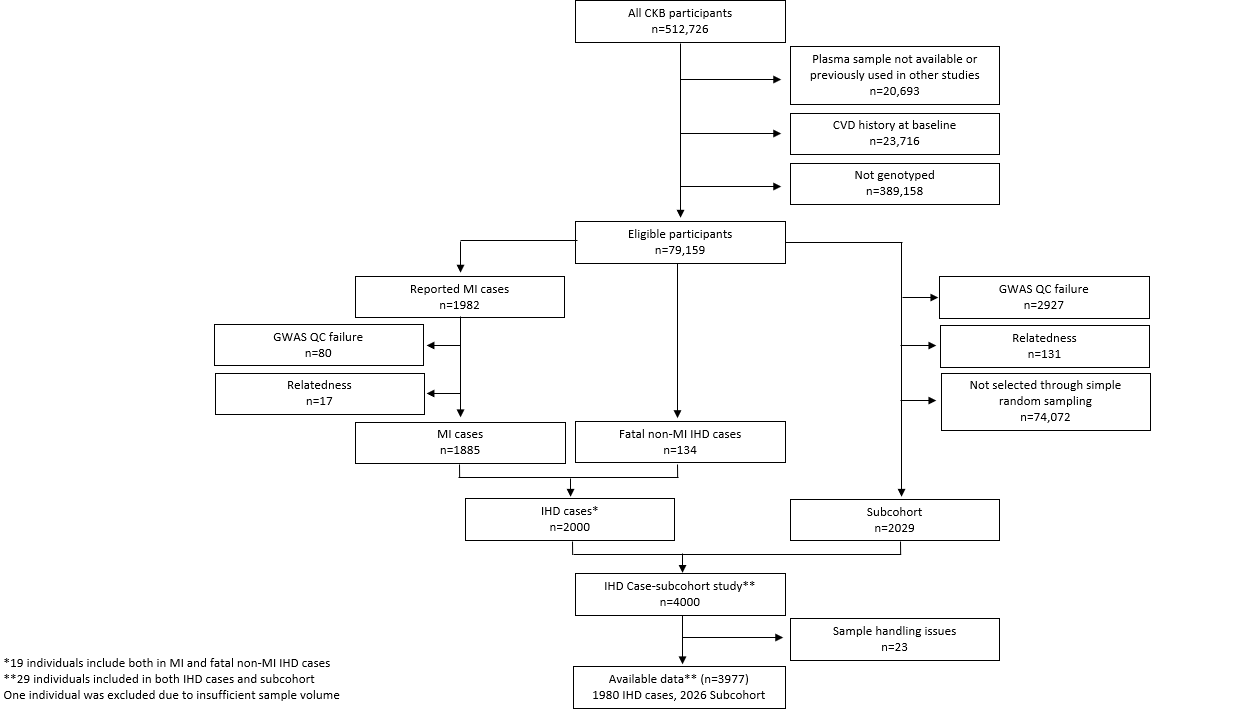


# eFigure 2. Cumulative variance explained by principal components for SomaScan protein biomarkers and baseline characteristics

The cumulative variance explained (%) is plotted against the number of principal components (red curve). The individual variance explained by each component is represented by bar chart. The blue dashed line indicates the point at which 90% of the variance is reached.

Abbreviation: PC: principal component


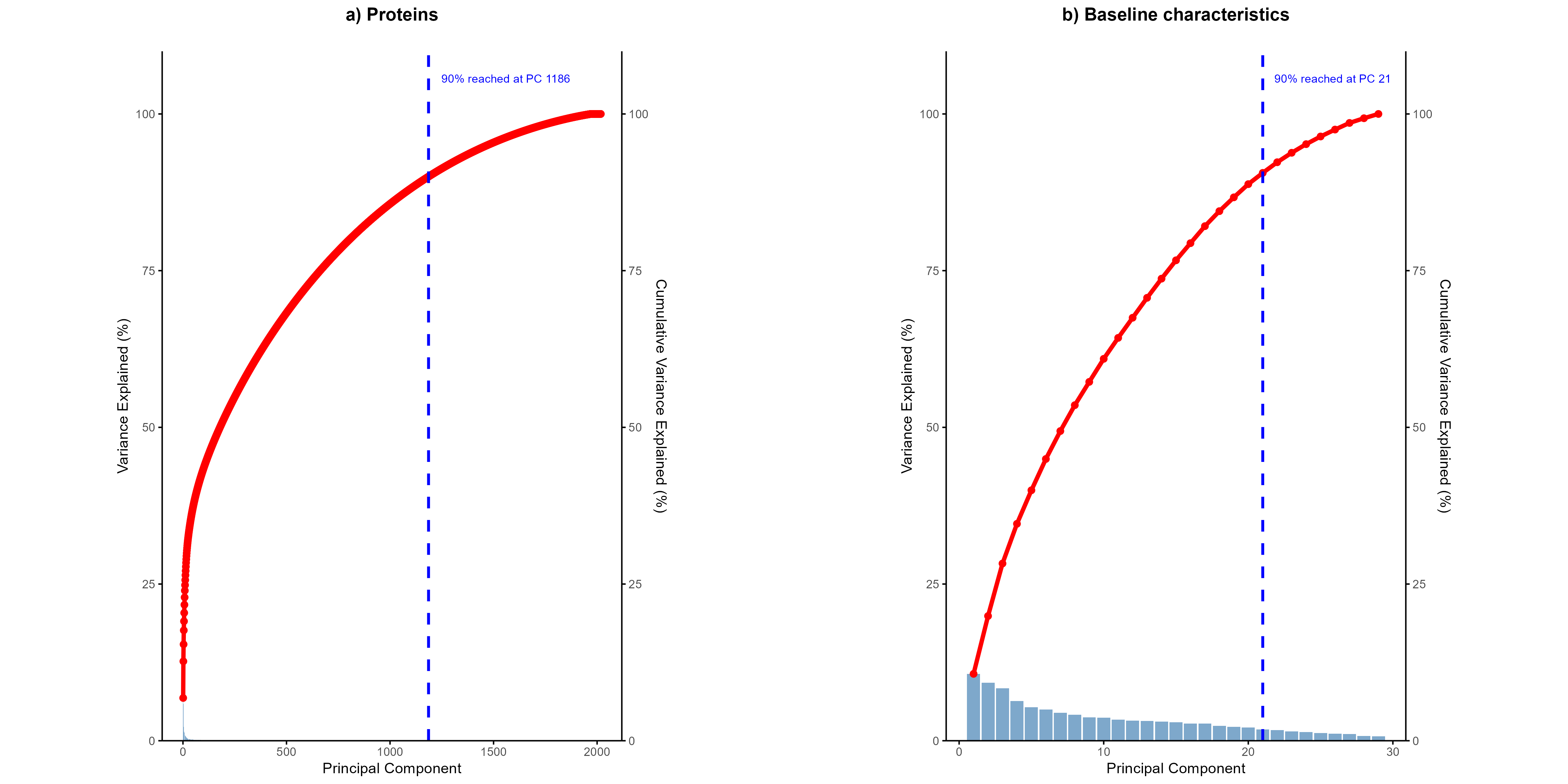


# eFigure 3. Exposure profiles by characteristics type of the top 25 SomaScan protein biomarkers with most positive and negative associations, overall and by sex

The bar plots show the number of baseline characteristics positively and negatively associated with the most frequently associated protein biomarkers after Bonferroni corrected p-value. The analyses are presented separately for females, males, and the overall. The 25 protein biomarkers with the most negative associations and the 25 protein biomarkers with the most positive associations are presented. The x-axis represents the protein biomarkers, while the y-axis indicates the number of baseline characteristics associated with each protein (left: negatively- and right: positively-associated). Bars are color-coded to represent different baseline characteristic groups. Analyses performed based on ANML data. Analyses are adjusted for age, age^2^, sex, study area, fasting time, fasting time^2^, outdoor temperature outdoor temperature^2^, and plate ID where appropriate. Abbreviations: ANML: Adaptive normalisation by maximum likelihood


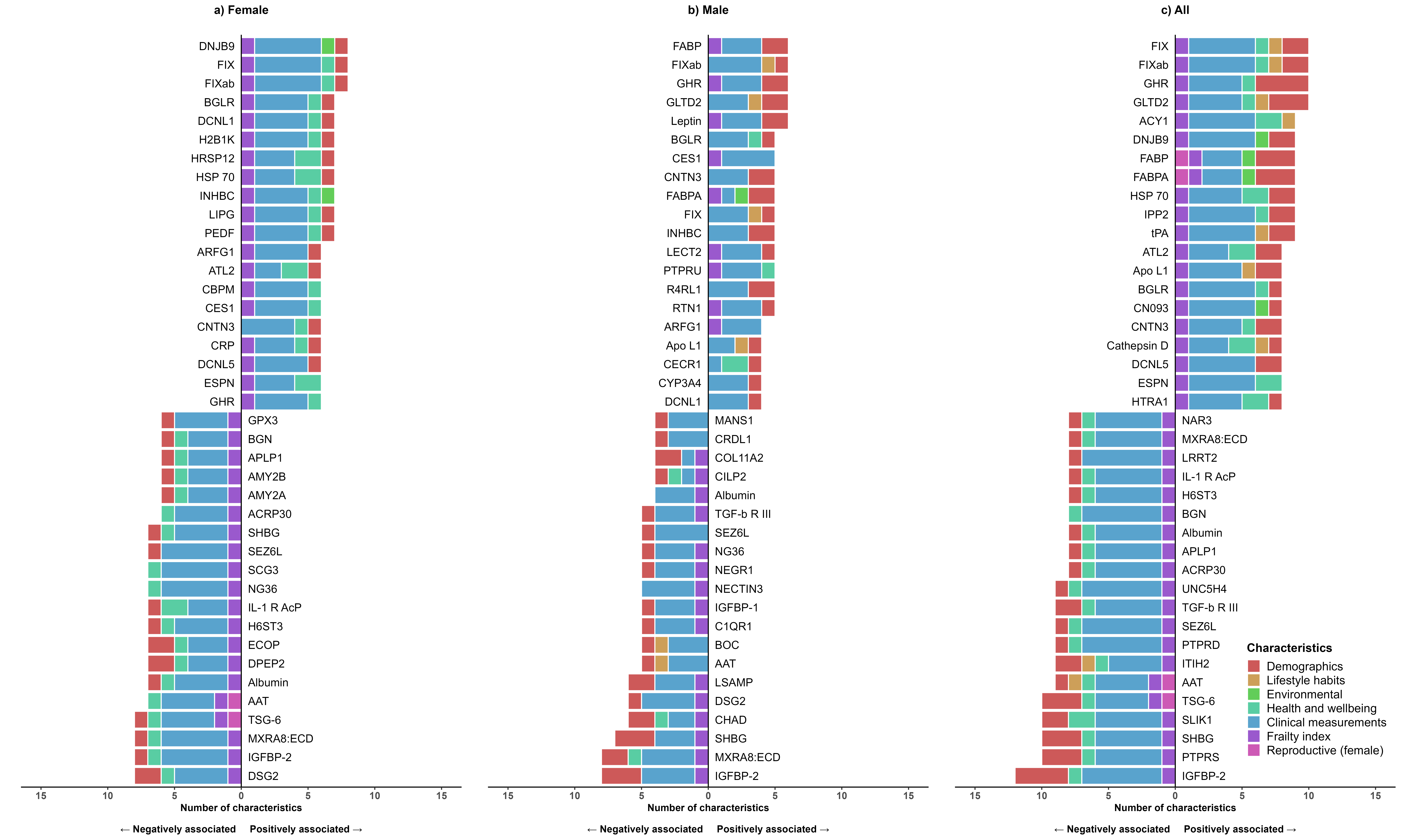


# eFigure 4. Associations of selected baseline characteristics with SomaScan protein biomarkers by sex

Analyses performed based on ANML data. Abbreviations: ANML: Adaptive normalisation by maximum likelihood; HBsAg+: Hepatitis B surface antigen seropsitive; SBP: systolic blood pressure; RPG: Random plasma glucose


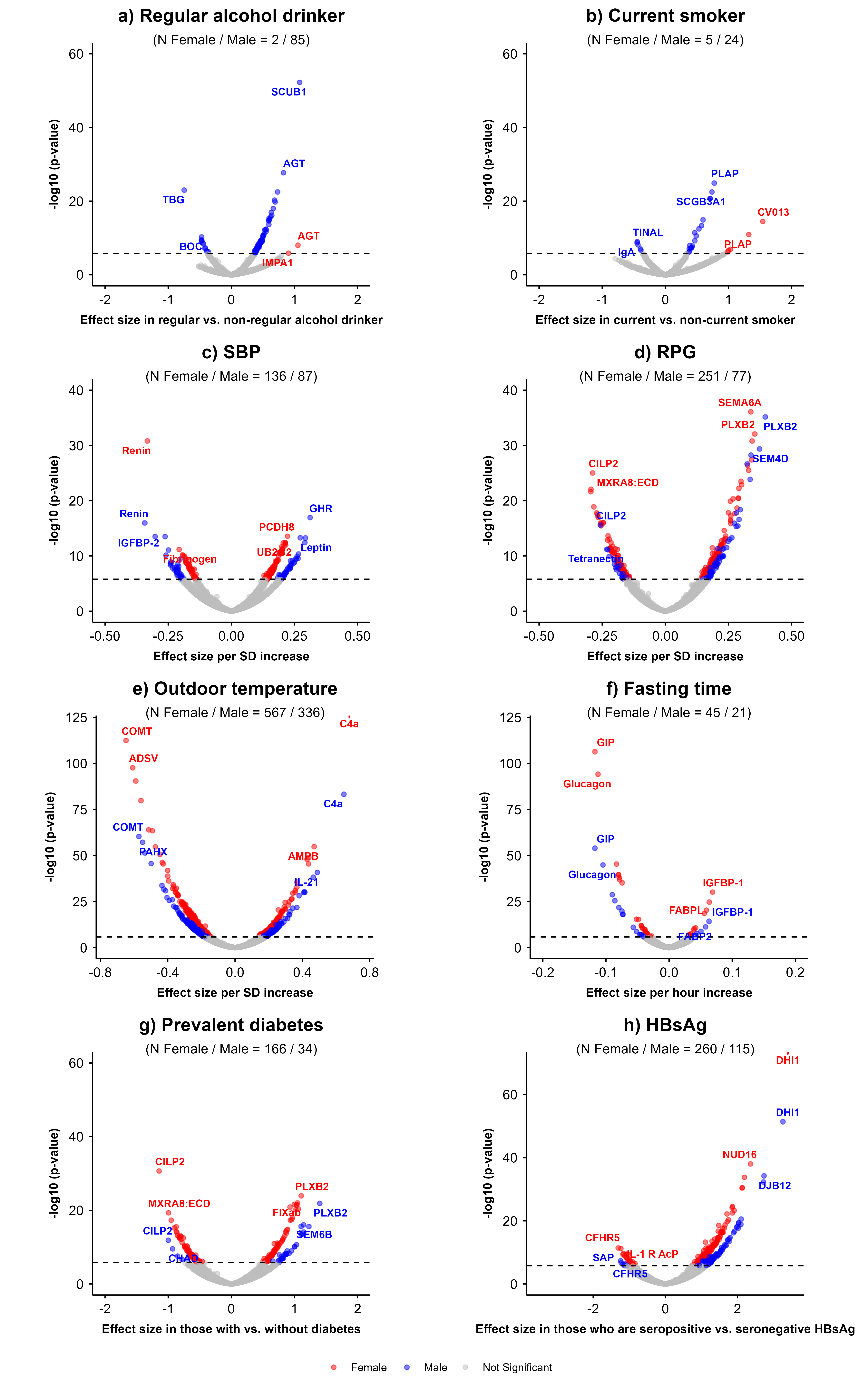


# eFigure 5. Comparison of associations of selected baseline characteristics and SomaScan protein biomarkers in female and male

Analyses performed based on ANML data. Abbreviations: ANML: Adaptive normalisation by maximum likelihood; BMI: Body mass index; HBsAg+: Hepatitis B surface antigen seropositive; SBP: systolic blood pressure; RPG: Random plasma glucose


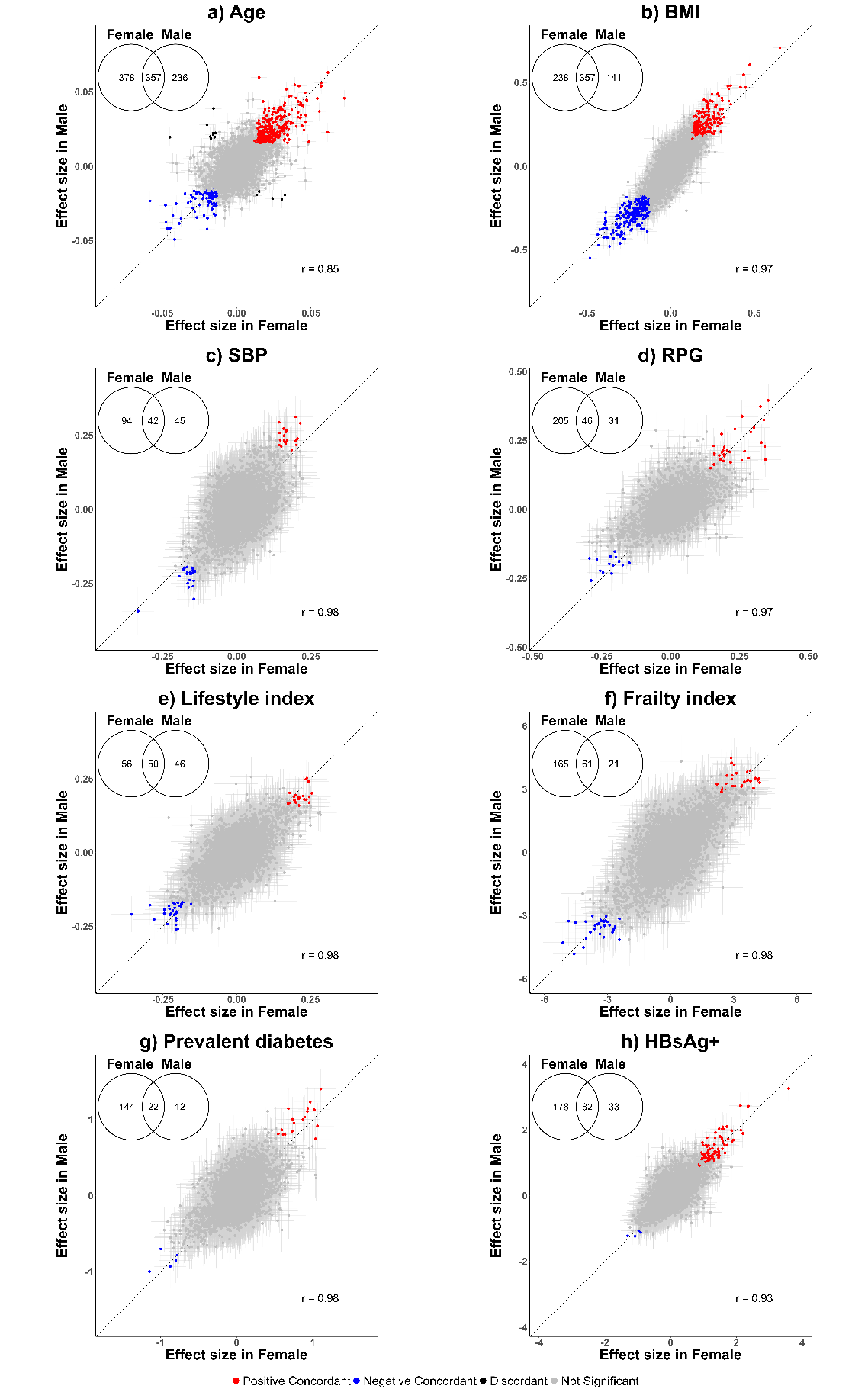


# eFigure 6. Age-associated protein biomarkers and their associations with other exposures by sex

Figures (i)a and (ii)a represent the associations of age in female and male, respectively, with protein biomarkers. The x-axis represents the effect size of the association between age and the protein biomarkers, while the y-axis indicates the –log10 p-value. Red dots denote positive Bonferroni corrected associations, blue dots denote negative Bonferroni corrected associations, and grey dots denote non-significant associations. Figures (i)b and (ii)b illustrate the top age-associated protein biomarkers in female and male, respectively, and their associations with other exposures. The width of the ribbons is inversely proportional to the p-value, indicating the strength of the association (smaller p-values correspond to wider ribbons). The colors of the ribbons represent different baseline characteristic groups. The top protein biomarkers that are not associated with other exposures are not presented in the figure. Analyses performed based on ANML data. Analyses are adjusted for age, age^2^, sex, study area, fasting time, fasting time^2^, outdoor temperature, outdoor temperature^2^ and plate ID, where appropriate.

Abbreviations: ANML: Adaptive normalisation by maximum likelihood; BMI: Body mass index; CO: carbon-monoxide; DBP: Diastolic blood pressure; HBsAg: Hepatitis B surface antigen virus; RPG: random plasma glucose


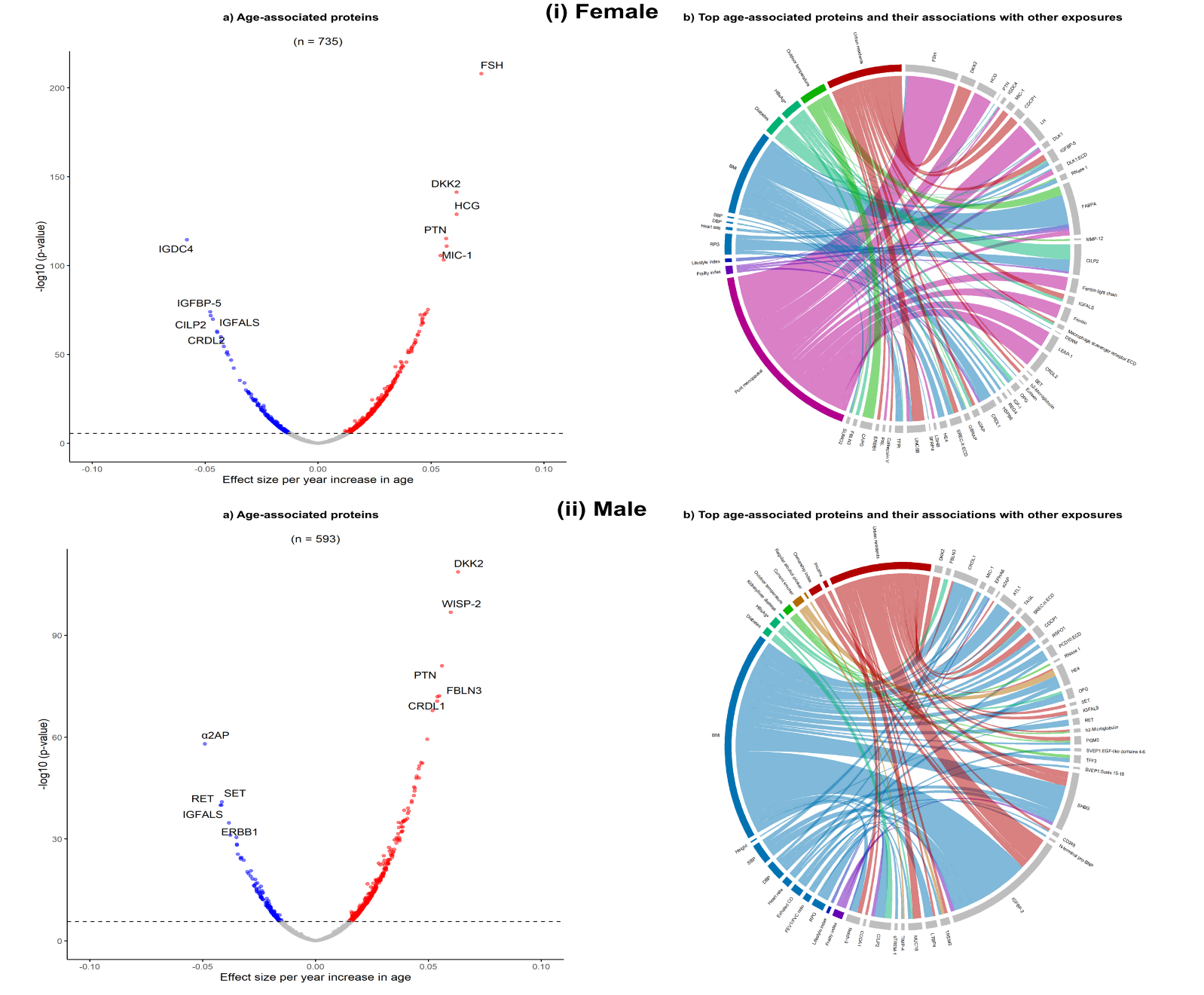


# eFigure 7. Post-menopause-associated protein biomarkers and their exposome associations, in females

Legend conventions as eFigure 5.

~~
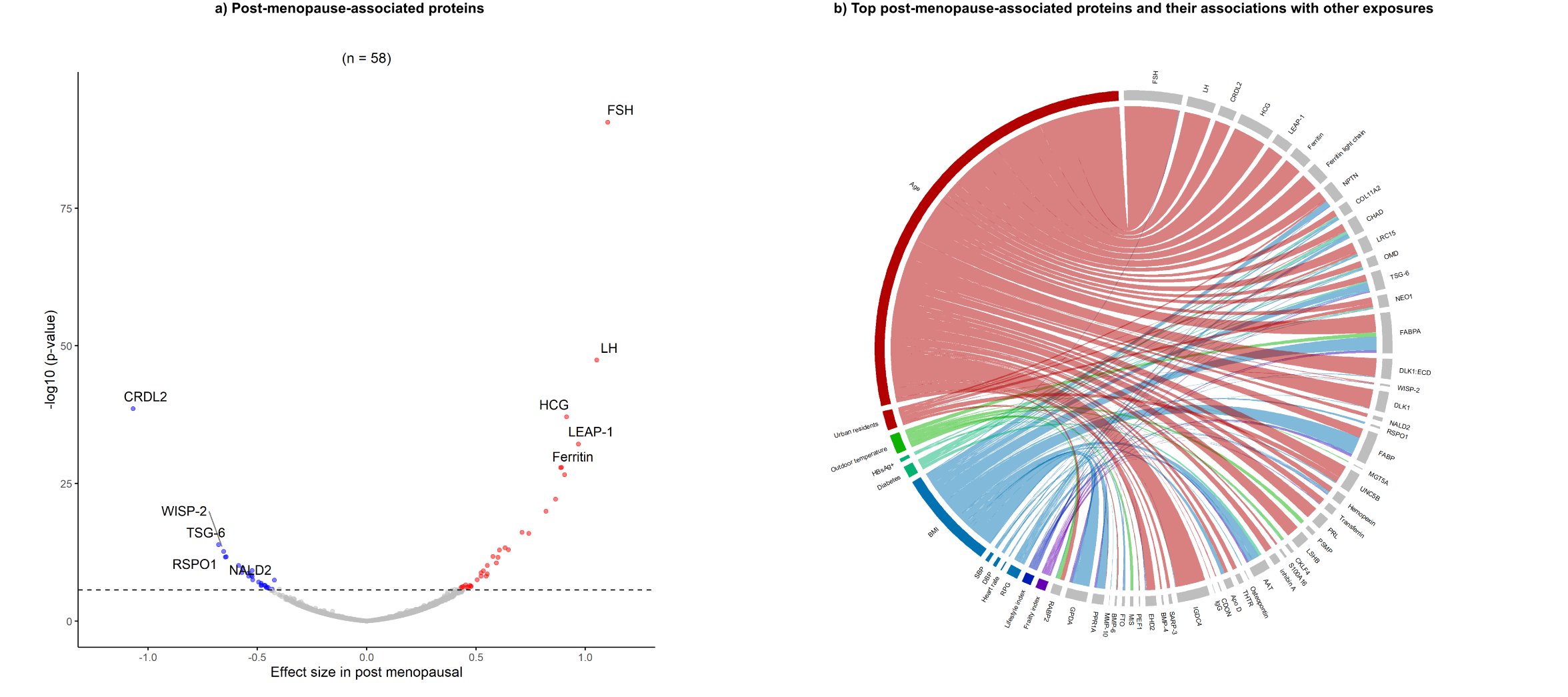
~~

# eFigure 8. Associations of selected baseline characteristics with protein biomarkers

Analyses performed based on ANML data. Abbreviations: ANML: Adaptive normalisation by maximum likelihood; HBsAg+: Hepatitis B surface antigen seropositive; SBP: systolic blood pressure; RPG: Random plasma glucose


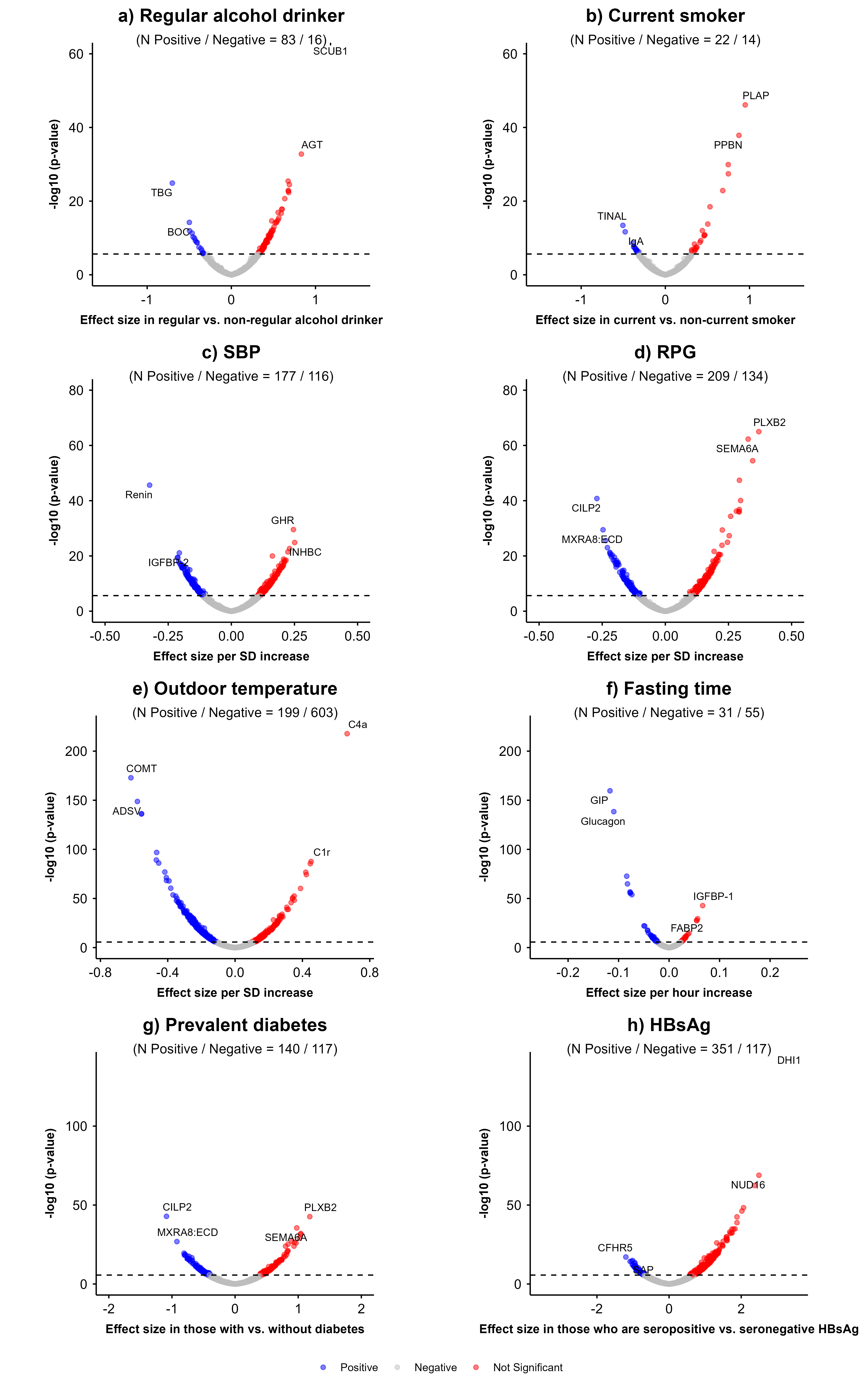


# eFigure 9. BMI-associated protein biomarkers and their associations with other exposures, in overall analyses

Legend conventions as eFigure 5.

~~
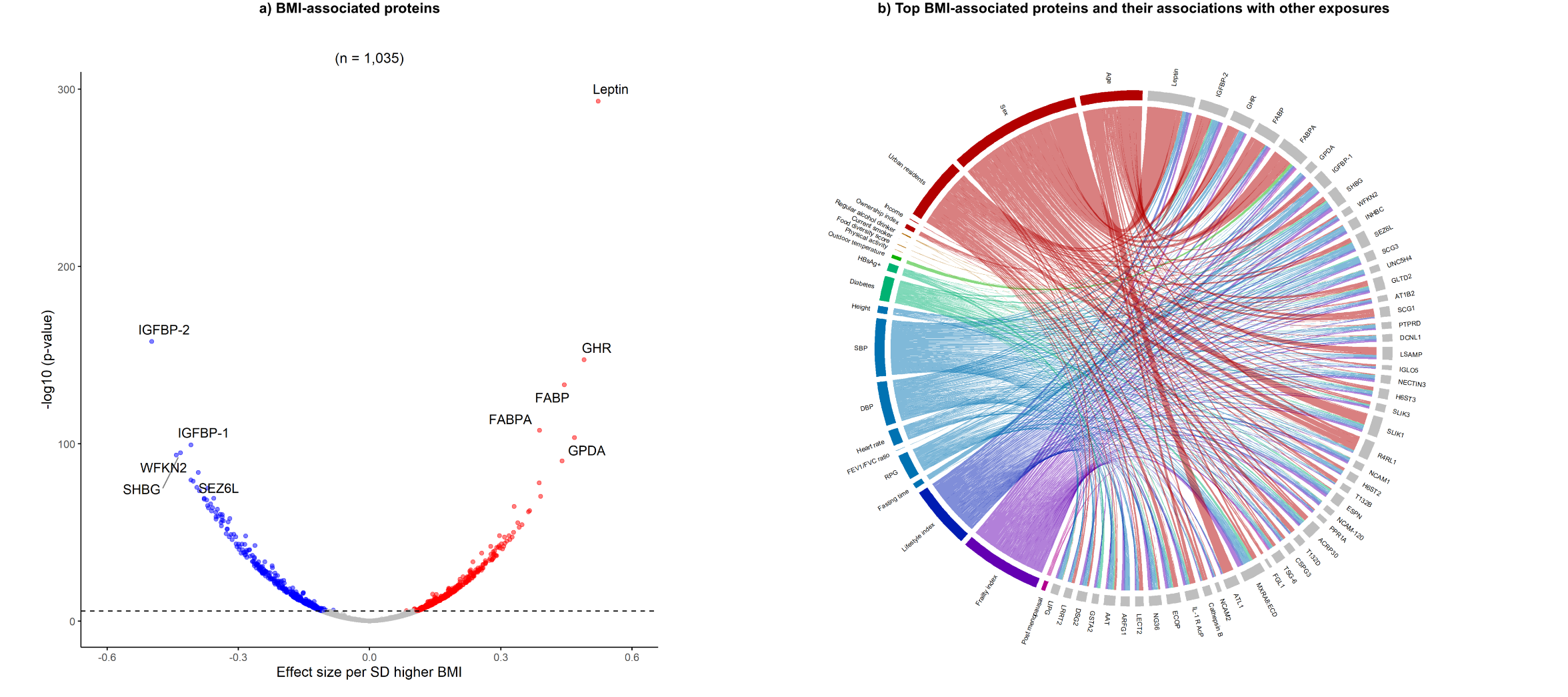
~~

# eFigure 10. BMI-associated protein biomarkers and their associations with other exposures, by sex

Legend conventions as eFigure 5.

~~
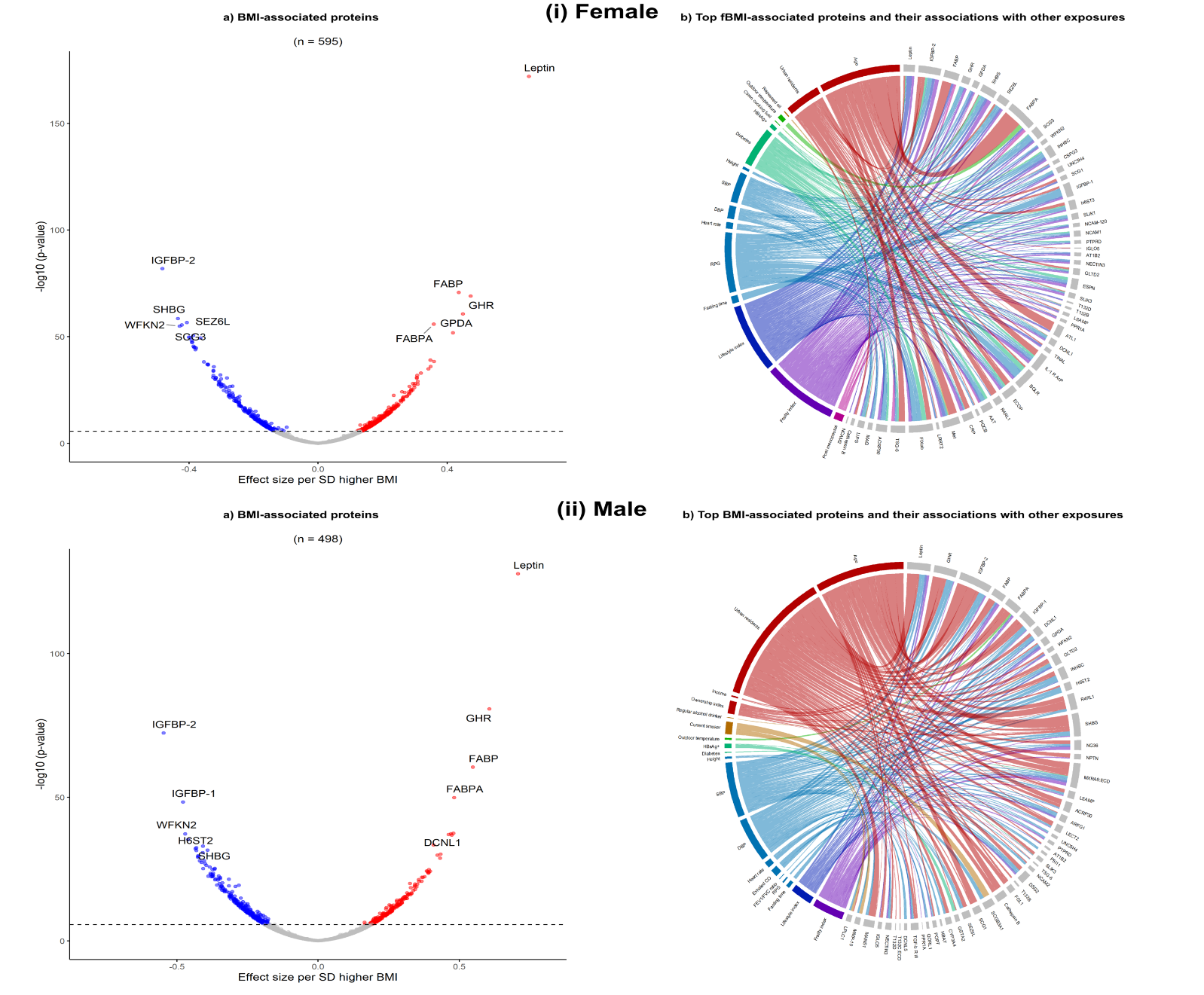
~~

# eFigure 11: Exposure profiles of 6597 SomaScan protein biomarkers by abundance level, in normalized dataset


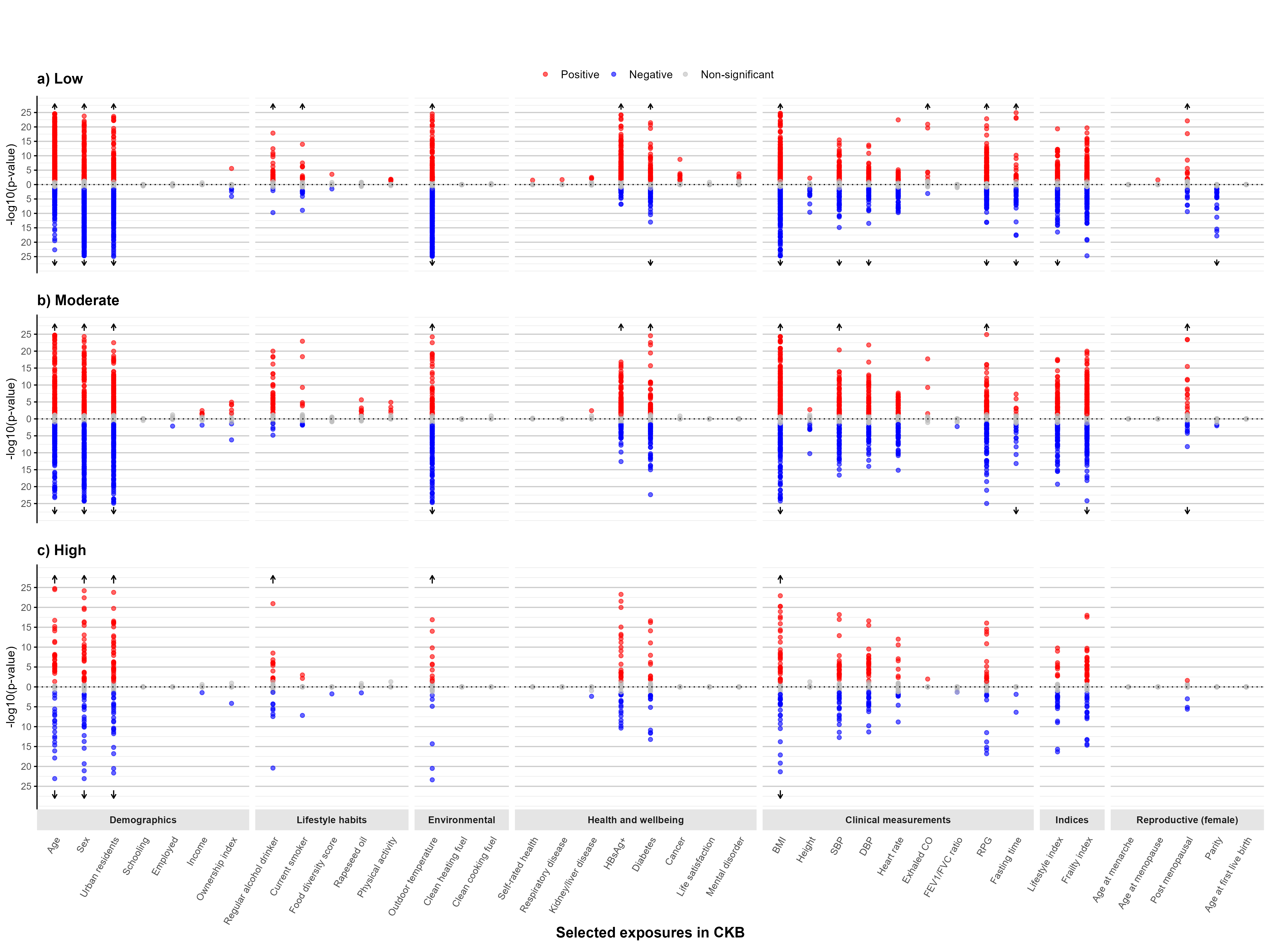


# eFigure 12. Exposure profiles of 6597 SomaScan protein biomarkers overall and by sex, in non-normalized dataset


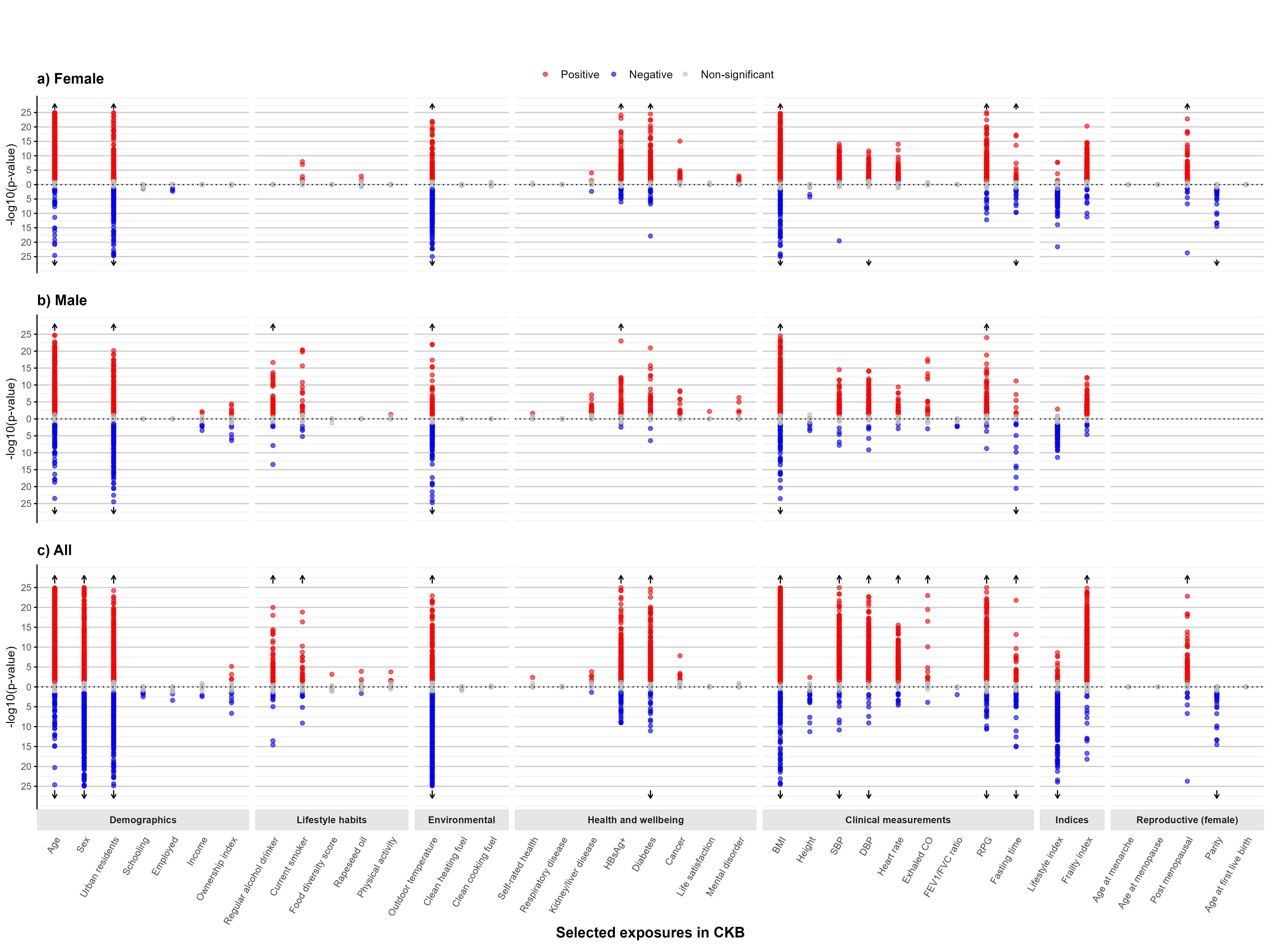


# eFigure 13. Comparison of associations of selected baseline characteristics and protein biomarkers in normalized and non-normalized datasets


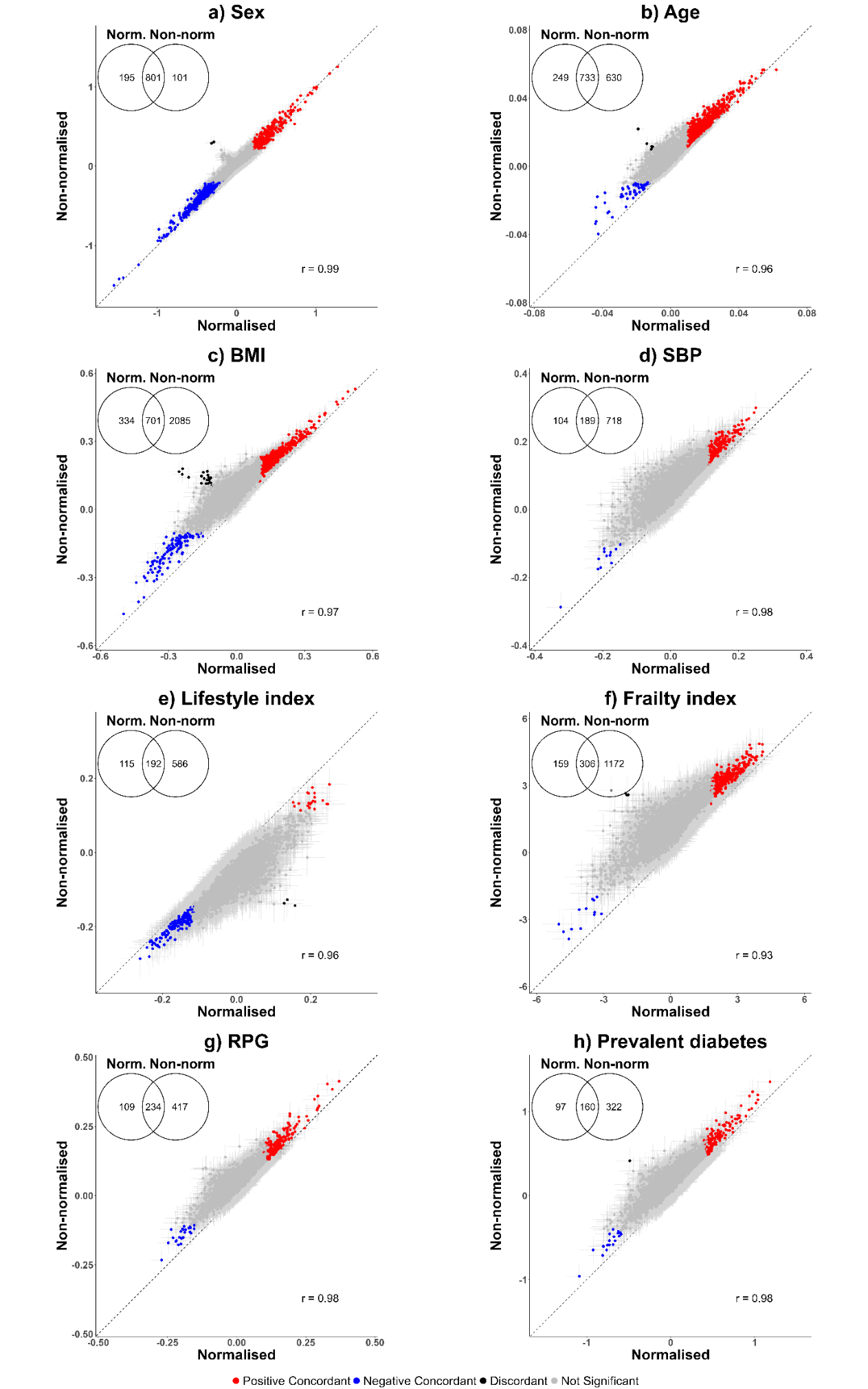


# eFigure 14. Exposure profiles of 6597 SomaScan and 2923 Olink protein biomarkers by platform and normalization status (SomaScan)


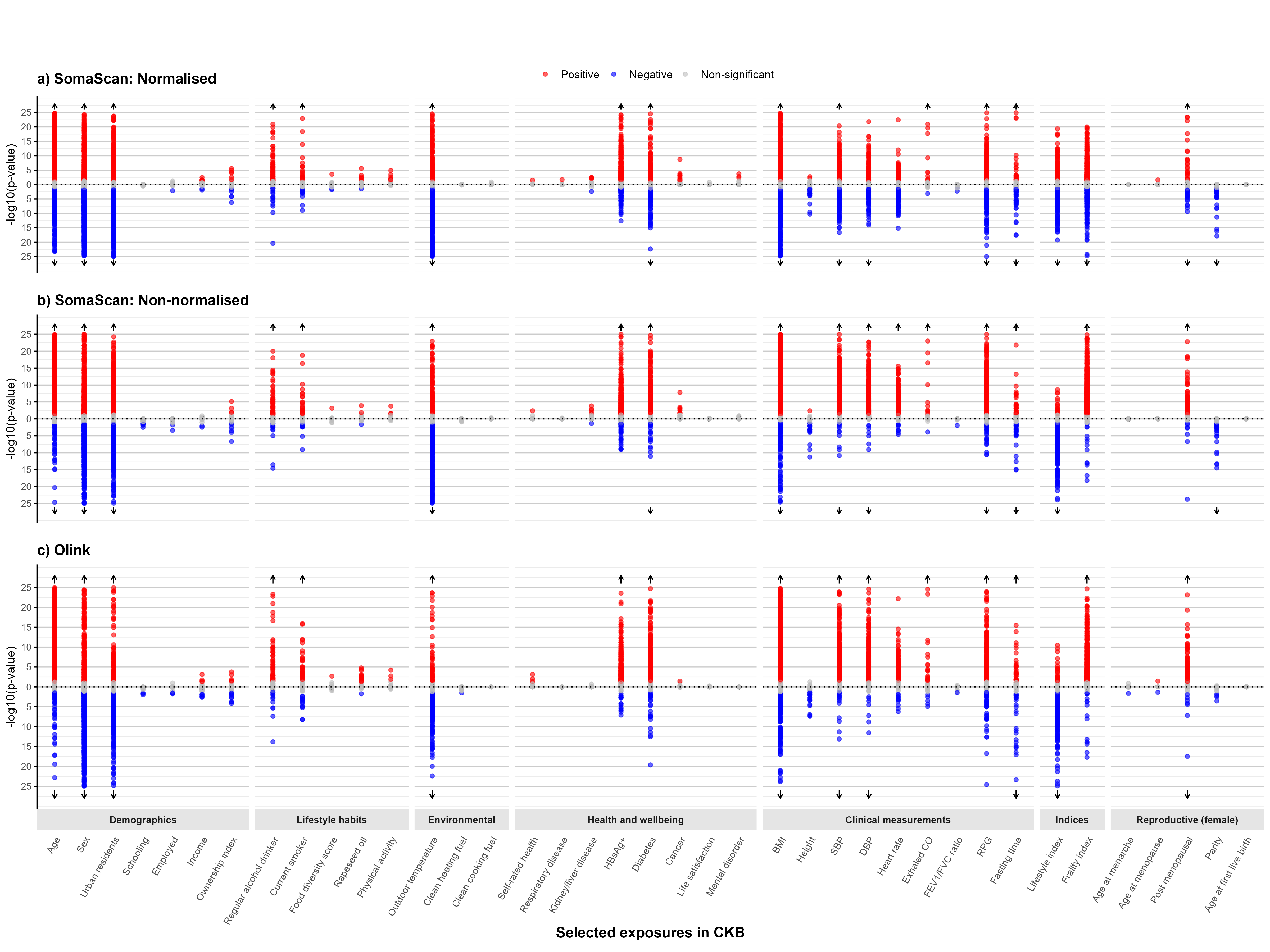


# References

1. Lv J, Yu C, Guo Y, Bian Z, Yang L, Chen Y, et al. Adherence to a healthy lifestyle and the risk of type 2 diabetes in Chinese adults. Int J Epidemiol. 2017;46(5):1410-20.

2. Sun Q, Yu D, Fan J, Yu C, Guo Y, Pei P, et al. Healthy lifestyle and life expectancy at age 30 years in the Chinese population: an observational study. Lancet Public Health. 2022;7(12):e994-e1004.

3. Fan J, Yu C, Guo Y, Bian Z, Sun Z, Yang L, et al. Frailty index and all-cause and cause-specific mortality in Chinese adults: a prospective cohort study. Lancet Public Health. 2020;5(12):e650-e60.
